# Supplementary material for: Performance of ChatGPT in Pediatric Audiology as Rated by Students and Experts
Source: J Clin Med. 2025 Jan 28;14(3):875. doi: 10.3390/jcm14030875 (PMC11818674; doi:10.3390/jcm14030875)
Supplement: Supplementary file 1 [file jcm-14-00875-s001.zip › jcm-3326457-supplementary.pdf]

SUPPLEMENTARY MATERIALS

Questions to ChatGPT in Polish (original version)..1

Questions with ChatGPT answers in Polish (original version)..2

Questions to ChatGPT in English language version 14

Questions with ChatGPT answers in English language version 15

ChatGPT's answers (to questions posed in English), translated into Polish using DeepL (note that the questions are the same as in the original Polish version) 31

Questions to ChatGPT in Polish (original version)

1. Czym jest system CROS?
2. Czym jest niedosłuch przewodzeniowy?
3. Jakie częstotliwości odpowiadają za odbiór dźwięków mowy u dzieci?
4. Jakie mogą być przyczyny zapalenia ucha u dziecka?
5. Jak zinterpretować badanie audiometrii tonalnej, czym jest norma słyszenia?
6. Jak wygląda badanie ABR u dziecka?
7. Na czym polegają przesiewowe badania słuchu u noworodków?
8. W jaki sposób przebiega wysiękowe zapalenie ucha środkowego u dziecka?
9. W jaki sposób można zbadać słuch u rocznego dziecka?
10. Na czym polega badanie audiometrii słownej?
11. Jakie typy tympanogramów wyróżniamy w audiometrii impedancyjnej?
12. W jakim celu wykonuje się wycisk ucha u dzieci?
13. Jak w audiogramie oznaczyć przewodnictwo powietrzne?
14. Co należy zrobić w przypadku nieprawidłowego wyniku przesiewowych badań słuchu u noworodka?
15. Co oznaczają żółte i niebieskie certyfikaty w książeczce zdrowia dziecka w kontekście przesiewowych badań słuchu dzieci w Polsce?
16. Jakie są przeciwwskazania do używania aparatów słuchowych na przewodnictwo powietrzne?
17. Jakie są najskuteczniejsze metody leczenia wysiękowego zapalenia ucha środkowego u dziecka?
18. Jak wygląda wynik badania audiometrii słownej i jak go interpretować?
19. Jakie są wyniki otoskopii w przypadku otosklerozy?
20. Jakie wszczepialne protezy słuchu można stosować u dzieci?

Questions with ChatGPT answers in Polish (original version)

1. Czym jest system CROS?

System CROS (Contralateral Routing of Signal) jest rodzajem aparatu słuchowego przeznaczonym dla osób z jednostronną głuchotą lub bardzo słabym słuchem w jednym uchu, przy jednoczesnym dobrym słuchu w drugim uchu. System CROS został zaprojektowany, aby przekierowywać dźwięki z uszkodzonego ucha na zdrowe ucho, co umożliwia lepsze zrozumienie mowy i dźwięków otoczenia.

System składa się z dwóch części: nadajnika umieszczonego na uszach z utratą słuchu i odbiornika na zdrowym uchu. Nadajnik zbiera dźwięki z otoczenia po stronie słabszego ucha i bezprzewodowo przekazuje je do odbiornika. Odbiornik odtwarza dźwięki, dzięki czemu użytkownik może słyszeć dźwięki z obu stron.

CROS jest skutecznym rozwiązaniem dla osób z jednostronną głuchotą, ponieważ pomaga w lokalizacji dźwięków i zrozumieniu mowy w hałaśliwym środowisku, poprawiając jakość życia. Istnieją różne rodzaje systemów CROS, w tym cyfrowe i analogowe, a także modele, które można indywidualnie dopasować do potrzeb użytkownika.

2. Czym jest niedosłuch przewodzeniowy?

Niedosłuch przewodzeniowy jest rodzajem utraty słuchu, który występuje, gdy dźwięk nie jest prawidłowo przekazywany przez zewnętrzny i środkowy ucho do wewnętrznego ucha. Problem ten różni się od niedosłuchu czuciowo-nerwowego, w którym uszkodzenie występuje w wewnętrznym uchu lub w nerwie słuchowym.

Przyczyny niedosłuchu przewodzeniowego mogą obejmować:

Zablokowanie przewodu słuchowego zewnętrznego: Może to być spowodowane przez nagromadzenie woskowiny, ciała obce w uchu lub zapalenie skóry przewodu słuchowego.

Problemy z błoną bębenkową: Uszkodzenie, perforacja (dziura) lub zbliznowacenie błony bębenkowej mogą wpływać na jej zdolność do efektywnego wibracji w odpowiedzi na dźwięk.

Zaburzenia ucha środkowego: Mogą to być na przykład otoskleroza (zaburzenie, które wpływa na kosteczki słuchowe) lub zapalenie ucha środkowego, które może prowadzić do gromadzenia się płynu i zmniejszać ruchomość kosteczek słuchowych.

Objawy niedosłuchu przewodzeniowego często obejmują zmniejszoną zdolność do słyszenia cichych dźwięków oraz trudności w rozumieniu mowy, szczególnie w hałaśliwym otoczeniu. Czasem osoby z tym typem niedosłuchu zauważają, że ich własny głos brzmi głośniej lub bardziej wyraźnie w uszkodzonym uchu.

Leczenie niedosłuchu przewodzeniowego zależy od jego przyczyny. Może obejmować usunięcie woskowiny, leczenie infekcji, operację naprawczą błony bębenkowej lub kosteczek słuchowych, a w niektórych przypadkach stosowanie aparatów słuchowych. W wielu przypadkach niedosłuch przewodzeniowy jest odwracalny, ale ważne jest, aby skonsultować się z lekarzem w celu właściwej diagnozy i leczenia.

3. Jakie częstotliwości odpowiadają za odbiór dźwięków mowy u dzieci?

Zakres częstotliwości, które są kluczowe dla odbioru mowy, jest podobny zarówno u dzieci, jak i u dorosłych. Mowa ludzka obejmuje szeroki zakres częstotliwości, jednak najważniejsze dla zrozumienia mowy są te w przedziale od około 250 Hz do 4000 Hz. Większość zrozumiałych informacji w mowie, takich jak samogłoski i niektóre spółgłoski, mieści się w niższej części tego zakresu, natomiast wyższe

częstotliwości są niezbędne do rozróżnienia między spółgłoskami, które brzmią podobnie, takimi jak "s" i "f" czy "p" i "t".

Zrozumienie mowy u dzieci jest niezwykle ważne dla rozwoju językowego i komunikacyjnego. U dzieci, które uczą się mówić i rozumieć język, nawet lekka utrata słuchu, zwłaszcza w zakresie wyższych częstotliwości, może wpłynąć na zdolność do nauki i korzystania z języka. Dlatego ważne jest, aby na wczesnym etapie wykrywać i leczyć wszelkie problemy ze słuchem.

W kontekście edukacji i rozwoju dziecka, oprócz częstotliwości mowy, ważne jest również zwracanie uwagi na jakość dźwięku i poziom szumu w otoczeniu, które mogą wpływać na zdolność dziecka do słuchania i zrozumienia mowy.

4. Jakie mogą być przyczyny zapalenia ucha u dziecka?

Zapalenie ucha u dzieci jest częstym problemem zdrowotnym i może mieć różne przyczyny. Oto niektóre z najczęstszych czynników prowadzących do zapalenia ucha u dzieci:

Infekcje wirusowe lub bakteryjne: Często zapalenie ucha występuje po infekcjach dróg oddechowych, takich jak przeziębienie lub grypa. Bakterie lub wirusy mogą przenikać do ucha środkowego, powodując zapalenie.

Niedojrzałość układu odpornościowego: Dzieci mają mniej dojrzały układ odpornościowy niż dorośli, co czyni je bardziej podatnymi na infekcje, w tym te dotyczące ucha.

Struktura anatomiczna: Krótsze i bardziej poziome trąbki Eustachiusza u dzieci ułatwiają przenikanie zarazków z nosogardzieli do ucha środkowego.

Alergie: Alergie mogą prowadzić do zapalenia i zablokowania trąbki Eustachiusza, co z kolei może prowadzić do zapalenia ucha.

Ekspozycja na dym tytoniowy i zanieczyszczenia: Dzieci, które są narażone na dym tytoniowy lub inne zanieczyszczenia środowiskowe, mają wyższe ryzyko zapalenia ucha.

Częste infekcje górnych dróg oddechowych: Dzieci, które często mają infekcje górnych dróg oddechowych, są bardziej narażone na zapalenie ucha środkowego.

Niedobory żywieniowe: Niedobory żywieniowe, szczególnie niedobór witaminy A, mogą osłabiać odporność, co zwiększa ryzyko infekcji, w tym zapalenia ucha.

Czynniki genetyczne: W niektórych przypadkach skłonność do zapalenia ucha może być dziedziczona.

Zwężenie nosa i gardła: Powiększone migdałki lub polipy nosa mogą przyczyniać się do problemów z wentylacją ucha środkowego.

Leczenie zapalenia ucha zależy od jego przyczyny i nasilenia. W wielu przypadkach stosuje się antybiotyki (w przypadku infekcji bakteryjnych), leczenie objawowe, a czasem wymagane jest drenaż ucha. Ważne jest, aby w przypadku podejrzenia zapalenia ucha skonsultować się z lekarzem.

5. Jak zinterpretować badanie audiometrii tonalnej, czym jest norma słyszenia?

Audiometria tonalna jest badaniem, które mierzy zdolność słyszenia różnych tonów (dźwięków) o różnych częstotliwościach (mierzonych w hercach, Hz) i głośnościach (mierzonych w decybelach, dB). Wyniki badania są zazwyczaj przedstawiane na wykresie zwanym audiogramem.

Oto jak interpretować wyniki audiometrii tonalnej:

Oś pozioma audiogramu: Reprezentuje częstotliwości (tony) od niskich (po lewej stronie, np. 250 Hz) do wysokich (po prawej stronie, np. 8000 Hz). Częstotliwości te odpowiadają różnym tonom, które można usłyszeć.

Oś pionowa audiogramu: Reprezentuje głośność dźwięku w decybelach (dB). Dźwięki na dole wykresu są cichsze, a te na górze głośniejsze.

Znaczniki na wykresie: Pokazują, przy jakiej głośności (dB) badana osoba zaczyna słyszeć dany ton (częstotliwość). Typowo, znaczniki te są różne dla lewego i prawego ucha.

Norma słyszenia: W typowym audiogramie norma słyszenia jest reprezentowana przez linie 20 dB lub niżej. Osoby ze słuchem w normie mogą słyszeć dźwięki poniżej tej linii. Utrata słuchu jest klasyfikowana jako lekka, umiarkowana, średnio ciężka, ciężka lub głęboka, w zależności od tego, przy jakiej głośności zaczynają słyszeć różne częstotliwości.

Typy utraty słuchu: Jeśli wszystkie znaczniki znajdują się powyżej linii normy słyszenia, oznacza to utratę słuchu. Jeśli linia dla niskich częstotliwości jest wyższa niż dla wysokich, może to wskazywać na utratę słuchu niskich tonów, a odwrotnie dla wysokich tonów.

Asymetria: Różnice między uszami mogą również być ważnymi informacjami. Asymetryczna utrata słuchu (różna w obu uszach) może wymagać dalszej diagnostyki.

Ważne jest, aby pamiętać, że interpretacja audiogramu powinna być przeprowadzana przez specjalistę w dziedzinie audiologii, który może właściwie zinterpretować wyniki w kontekście historii medycznej pacjenta, jego objawów i potrzeb. Audiometria tonalna dostarcza istotnych informacji o charakterze i stopniu utraty słuchu, co jest kluczowe dla odpowiedniego planowania leczenia, w tym doboru aparatów słuchowych lub innych interwencji.

6. Jak wygląda badanie ABR u dziecka?

Badanie ABR (ang. Auditory Brainstem Response) jest testem, który ocenia, jak dobrze sygnały dźwiękowe są przekazywane przez nerw słuchowy do pnia mózgu. Jest to szczególnie przydatne badanie u małych dzieci, w tym niemowląt, ponieważ nie wymaga świadomej reakcji od pacjenta. Badanie ABR jest często stosowane do oceny słuchu u noworodków i małych dzieci, którzy nie mogą jeszcze samodzielnie zgłaszać swoich doświadczeń słuchowych.

Oto, jak zwykle przebiega badanie ABR u dziecka:

Przygotowanie: Dziecko powinno być zrelaksowane lub śpiące podczas badania. W niektórych przypadkach, szczególnie u starszych dzieci, może być konieczne podanie lekkiego środka uspokajającego, aby zapewnić spokojny sen przez cały czas trwania testu.

Umieszczanie elektrod: Na skórze dziecka, zazwyczaj na głowie i za uszami, umieszcza się małe, klejące elektrody. Elektrody te rejestrują aktywność elektryczną w mózgu w odpowiedzi na dźwięk.

Podawanie dźwięków: Przez słuchawki umieszczone w uszach dziecka podawane są specyficzne dźwięki (zwykle kliknięcia lub tonalne pipy). Dźwięki te są prezentowane w różnych głośnościach, aby ocenić, przy jakim minimalnym poziomie głośności mózg zaczyna reagować.

Rejestrowanie odpowiedzi: Podczas gdy dźwięki są odtwarzane, elektrody rejestrują aktywność elektryczną w mózgu dziecka. Zapisywane są odpowiedzi pnia mózgu na te dźwięki.

Analiza danych: Dane zebrane przez elektrody są analizowane, aby określić, czy i jak dobrze dźwięk jest przetwarzany przez nerw słuchowy i mózg.

Bezpieczeństwo i komfort: Badanie ABR jest całkowicie bezbolesne i bezpieczne. Ważne jest, aby dziecko pozostało nieruchomo podczas badania, stąd konieczność snu lub lekkiego sedacji.

Wyniki badania ABR mogą pomóc w diagnozowaniu niedosłuchu i innych zaburzeń słuchu u dzieci. Są one cenne, ponieważ mogą dostarczyć informacji o słuchu dziecka, zanim będzie ono w stanie samodzielnie odpowiedzieć na testy słuchowe. Na podstawie wyników ABR można planować dalsze leczenie, w tym dobór aparatów słuchowych lub, w niektórych przypadkach, implantacji ślimakowej.

7. Na czym polegają przesiewowe badania słuchu u noworodków?

Przesiewowe badania słuchu u noworodków to proces, który ma na celu wczesne wykrycie ewentualnych problemów ze słuchem. Wykonywane są zwykle w pierwszych dniach życia dziecka, często jeszcze w szpitalu po urodzeniu. Celem tych badań jest jak najszybsze zidentyfikowanie noworodków, które mogą mieć niedosłuch, aby jak najszybciej rozpocząć odpowiednią interwencję i leczenie.

Przesiewowe badania słuchu u noworodków zazwyczaj obejmują jedną lub obie z następujących metod:

Otoemisyjne badanie przesiewowe (OAE, ang. Otoacoustic Emissions):

W tej metodzie do małego, miękkiego urządzenia umieszczonego w uchu dziecka wysyłane są dźwięki.

Urządzenie to mierzy następnie echo tych dźwięków odbijających się od ucha wewnętrznego (ślimaka).

Jeśli w uchu wewnętrznym nie występują otoemisje akustyczne (czyli nie dochodzi do generowania tych "ech"), może to wskazywać na problem ze słuchem.

ABR (Auditory Brainstem Response) – badanie odpowiedzi pnia mózgu na bodźce słuchowe:

Podczas tego badania na głowie dziecka umieszcza się małe elektrody, które rejestrują aktywność elektryczną w mózgu w odpowiedzi na dźwięk.

Test ABR jest szczególnie przydatny w identyfikacji głębszych form niedosłuchu, w tym niedosłuchu nerwowego.

Oba testy są szybkie, bezbolesne i mogą być wykonywane podczas snu dziecka. Nie wymagają aktywnej współpracy ze strony noworodka, co jest kluczowe w przypadku badań przesiewowych w tej grupie wiekowej.

Jeśli wyniki pierwszego przesiewowego badania słuchu są niepewne lub wskazują na potencjalny problem, zaleca się przeprowadzenie dodatkowych, bardziej szczegółowych badań audiologicznych. Wczesne wykrycie problemów ze słuchem jest niezwykle ważne, ponieważ pozwala na szybkie rozpoczęcie leczenia i wsparcia, co jest kluczowe dla prawidłowego rozwoju mowy i języka u dziecka.

8. W jaki sposób przebiega wysiękowe zapalenie ucha środkowego u dziecka?

Wysiękowe zapalenie ucha środkowego, znane również jako zapalenie ucha środkowego z wysiękiem, jest stanem, w którym za błoną bębenkową gromadzi się płyn, niekoniecznie związany z infekcją bakteryjną czy wirusową. Jest to częsta przyczyna problemów ze słuchem u dzieci. Oto, jak zwykle przebiega ten stan:

Początek: Wysiękowe zapalenie ucha środkowego może rozwinąć się po ostrej infekcji ucha środkowego, gdy płyn pozostaje w uchu środkowym po wyeliminowaniu infekcji. Może się również rozwinąć niezależnie, jako wynik dysfunkcji trąbki Eustachiusza, która odpowiada za wyrównywanie ciśnienia powietrza i odprowadzanie płynów z ucha środkowego.

Objawy: Utrata słuchu: Najbardziej zauważalnym objawem wysiękowego zapalenia ucha środkowego jest często zmniejszona zdolność słyszenia. Płyn w uchu środkowym tłumi dźwięki, co sprawia, że są one bardziej przytłumione. Uczucie pełności lub ciśnienia w uchu: Dzieci mogą skarżyć się na uczucie zatkanego ucha. Brak silnego bólu: W odróżnieniu od ostrego zapalenia ucha środkowego, wysiękowe zapalenie ucha środkowego rzadko powoduje intensywny ból. Problemy z równowagą: Czasami może

wystąpić lekkie zawroty głowy lub problemy z równowagą. Brak gorączki: Zwykle nie towarzyszy temu stanowi gorączka.

Diagnoza: Diagnozę stawia lekarz na podstawie badania otoskopowego, podczas którego ocenia wygląd błony bębenkowej i sprawdza, czy za nią znajduje się płyn. Możliwe jest również przeprowadzenie tympanometrii, która mierzy ruchomość błony bębenkowej, co może pomóc w potwierdzeniu obecności płynu.

Leczenie:

Wiele przypadków wysiękowego zapalenia ucha środkowego rozwiązuje się samoistnie. Lekarz może zalecić obserwację i kontrolne wizyty. W przypadku trwających problemów ze słuchem lub częstych nawrotów zapalenia, lekarz może zaproponować zastosowanie rurki wentylacyjnej. Rurka ta jest umieszczana w błonie bębenkowej podczas krótkiego zabiegu chirurgicznego i pozwala na wyrównanie ciśnienia oraz odprowadzanie płynu z ucha środkowego.

Monitorowanie i dalsza opieka:

Regularne wizyty kontrolne są ważne, aby monitorować stan słuchu dziecka oraz sprawdzać, czy płyn w uchu środkowym się wchłania. W przypadku utrzymujących się problemów ze słuchem lub mowy, może być konieczne skierowanie dziecka do specjalisty, takiego jak audiolog lub logopeda, dla dalszej oceny i ewentualnej terapii.

Prewencja:

Zapobieganie infekcjom górnych dróg oddechowych, które mogą prowadzić do wysiękowego zapalenia ucha środkowego, jest ważne, zwłaszcza w okresie jesienno-zimowym. Unikanie ekspozycji na dym tytoniowy i inne czynniki drażniące może również pomóc w zapobieganiu powstawaniu tego stanu.

Wysiękowe zapalenie ucha środkowego jest zwykle mniej dramatyczne niż ostre zapalenie ucha, ale ze względu na potencjalny wpływ na rozwój słuchu i mowy, ważne jest, aby było odpowiednio diagnozowane i leczone.

9. W jaki sposób można zbadać słuch u rocznego dziecka?

Badanie słuchu u rocznego dziecka wymaga specjalnych metod, ponieważ dzieci w tym wieku nie są w stanie świadomie współpracować w standardowych testach audiometrycznych, jakie stosuje się u starszych dzieci i dorosłych. Oto kilka metod, które mogą być użyte do oceny słuchu u rocznego dziecka:

Otoemisyjne badanie przesiewowe (OAE, ang. Otoacoustic Emissions Test): To nieinwazyjna metoda, która mierzy echo (otoemisje) wytwarzane przez zdrowe ucho wewnętrzne w odpowiedzi na dźwięki. Test jest szybki, bezbolesny i może być wykonany podczas snu dziecka.

Badanie odpowiedzi pnia mózgu na bodźce słuchowe (ABR, ang. Auditory Brainstem Response): Badanie ABR mierzy, jak nerw słuchowy i mózg reagują na dźwięk. Podczas testu na głowie dziecka umieszcza się elektrody, które rejestrują aktywność elektryczną w odpowiedzi na dźwięki emitowane przez słuchawki. Test jest bezbolesny i zwykle wymaga, aby dziecko spało lub było w stanie spokojnego wypoczynku.

Behawioralne testy audiometryczne: Testy behawioralne wymagają obserwacji reakcji dziecka na dźwięki. Może to obejmować:

Obserwację warunkową (ang. Visual Reinforcement Audiometry, VRA): W tej metodzie dziecko jest trenowane, aby obrócić głowę w kierunku dźwięku. Gdy dziecko reaguje na dźwięk, jest nagradzane wizualnie, na przykład poprzez pokazanie zabawki lub zapalenie świateł.

Testy zabawkowe (ang. Play Audiometry): Chociaż ta metoda jest zwykle stosowana u nieco starszych dzieci, może być również przystosowana do młodszych. Polega na uczynieniu reakcji na dźwięk częścią prostej gry.

Tympanometria: To badanie, które mierzy, jak dobrze błona bębenkowa wibruje w odpowiedzi na zmiany ciśnienia powietrza. Jest to przydatne w diagnozowaniu problemów z uchem środkowym, które mogą wpływać na słuch, takich jak wysiękowe zapalenie ucha środkowego.

Ważne jest, aby regularnie badać słuch dziecka, szczególnie jeśli istnieją jakiekolwiek obawy dotyczące jego rozwoju słuchu, mowy lub języka. Wczesne wykrycie i leczenie problemów ze słuchem jest kluczowe dla rozwoju komunikacyjnego dziecka.

10. Na czym polega badanie audiometrii słownej?

Audiometria słowna, znana również jako test rozumienia mowy, jest rodzajem badania słuchu, które ocenia, jak dobrze osoba rozumie mowę. Jest to ważny element oceny słuchu, ponieważ umożliwia lepsze zrozumienie, jak niedosłuch wpływa na zdolność komunikacji w codziennych sytuacjach. Oto, jak przebiega to badanie:

Przygotowanie: Osoba badana zazwyczaj nosi słuchawki lub jest umieszczona w dźwiękoszczelnym pomieszczeniu. W zależności od potrzeb, badanie może być przeprowadzone za pomocą głośników w otwartym polu, co jest szczególnie ważne dla osób używających aparatów słuchowych lub implantów ślimakowych.

Prezentacja słów: Podczas testu prezentowane są listy słów lub zdania. Słowa te są zazwyczaj dobrze znane i reprezentują typową mowę, aby wyniki były jak najbardziej miarodajne. Słowa mogą być prezentowane w różnych poziomach głośności, aby określić minimalny poziom, przy którym mowa jest zrozumiała.

Ocena rozumienia: Osoba badana jest proszona o powtórzenie każdego słyszanego słowa lub zdania. Wyniki są rejestrowane jako procent poprawnie zrozumianych i powtórzonych słów.

Interpretacja wyników: Wyniki audiometrii słownej są wyrażane procentowo i pokazują, jak dokładnie osoba rozumie mowę przy różnych poziomach głośności. Te wyniki pomagają zrozumieć, w jakim stopniu niedosłuch wpływa na zdolność rozumienia mowy.

Zastosowanie w leczeniu niedosłuchu: Wyniki audiometrii słownej są wykorzystywane do oceny skuteczności aparatów słuchowych lub implantów ślimakowych. Badanie pomaga również w dostosowaniu i kalibracji urządzeń słuchowych.

Audiometria słowna jest szczególnie ważna w ocenie, jak osoby z niedosłuchem radzą sobie w typowych sytuacjach komunikacyjnych oraz w planowaniu i monitorowaniu leczenia niedosłuchu.

11. Jakie typy tympanogramów wyróżniamy w audiometrii impedancyjnej?

W audiometrii impedancyjnej, która obejmuje tympanometrię, wyróżnia się kilka typów tympanogramów, które reprezentują różne charakterystyki ruchomości błony bębenkowej i ucha środkowego w odpowiedzi na zmiany ciśnienia. Oto podstawowe typy tympanogramów:

Typ A:

A: Jest to normalny tympanogram, wskazujący na normalną ruchomość błony bębenkowej i ucha środkowego. Charakteryzuje się wyraźnym szczytem, który wskazuje na optymalne ciśnienie w uchu środkowym.

A(S) lub A shallow: Oznacza mniejszą niż normalna ruchomość błony bębenkowej. Może to być związane z większą sztywnością systemu, np. w przypadku otosklerozy.

A(D) lub A deep: Wskazuje na zwiększoną ruchomość błony bębenkowej i/lub ucha środkowego, co może wystąpić w przypadkach, takich jak przerwanie łańcucha kosteczek słuchowych.

Typ B:

Tympanogram typu B jest płaski, co wskazuje na brak zmian ruchomości w odpowiedzi na zmiany ciśnienia. Może to być spowodowane obecnością płynu w uchu środkowym (jak w przypadku wysiękowego zapalenia ucha środkowego), perforacją błony bębenkowej lub zatkanym kanałem słuchowym zewnętrznym (np. przez woskowinę).

Typ C:

Tympanogram typu C ma szczyt, który jest przesunięty w kierunku ujemnych ciśnień. Wskazuje to na ujemne ciśnienie w uchu środkowym, co jest często związane z niedrożnością trąbki Eustachiusza. Może to wystąpić na przykład po infekcji górnych dróg oddechowych.

Tympanogramy są użytecznym narzędziem w diagnozowaniu różnych stanów ucha środkowego. Dostarczają one informacji nie tylko o obecności ewentualnego płynu w uchu środkowym, ale również o stanach, które mogą wpływać na ruchomość błony bębenkowej i ciśnienie w uchu środkowym.

12. W jakim celu wykonuje się wycisk ucha u dzieci?

Wycisk ucha u dzieci wykonuje się głównie w celu stworzenia indywidualnie dopasowanego ucha zewnętrznego dla aparatów słuchowych, protez słuchowych (np. implantów ślimakowych), ochronników słuchu lub wkładek przeciwhałasowych. Oto główne cele i zastosowania wycisku ucha:

Indywidualne Dopasowanie Aparatów Słuchowych: Aby aparat słuchowy działał efektywnie i był wygodny, musi być idealnie dopasowany do kanału ucha dziecka. Wycisk ucha pozwala na stworzenie indywidualnej formy, która jest podstawą do wykonania wkładki ucha lub tzw. muszli aparatu słuchowego.

Optymalizacja Przenoszenia Dźwięku: Indywidualnie dopasowane wkładki zapewniają lepsze przenoszenie dźwięku, minimalizując straty i poprawiając jakość słyszenia.

Komfort i Stabilność: Dzieci są bardzo ruchliwe, dlatego ważne jest, aby aparat słuchowy lub wkładka przeciwhałasowa była stabilna i bezpieczna w uchu. Indywidualne dopasowanie zapewnia większy komfort i zmniejsza ryzyko wypadnięcia aparatu z ucha.

Ochrona Przed Hałasem: W przypadku dzieci, które potrzebują ochrony słuchu (np. w hałaśliwym środowisku), wycisk ucha może być użyty do stworzenia indywidualnych ochronników słuchu.

Zastosowanie w Implantach Ślimakowych: Dla dzieci z implantami ślimakowymi wycisk ucha może być potrzebny do wykonania odpowiedniego mocowania zewnętrznej części urządzenia.

Zapobieganie Problematom z Uchem Środkowym: W niektórych przypadkach, na przykład przy częstych infekcjach ucha środkowego, indywidualnie dopasowane wkładki mogą być wykorzystywane do ochrony ucha przed wodą.

Wycisk ucha wykonuje się w specjalistycznych warunkach przez audiologa lub innego specjalistę. Procedura ta jest zwykle szybka i bezbolesna, ale wymaga współpracy dziecka. Wycisk jest następnie używany do stworzenia dokładnego odlewu kanału ucha, na podstawie którego produkuje się indywidualne rozwiązanie dopasowane do potrzeb słuchowych i ochronnych dziecka.

13. Jak w audiogramie oznaczyć przewodnictwo powietrzne?

W audiogramie przewodnictwo powietrzne jest oznaczane za pomocą specyficznych symboli, które reprezentują poziom słyszenia w odniesieniu do różnych częstotliwości dźwięku, mierzonego w decybelach (dB). Przewodnictwo powietrzne odnosi się do zdolności ucha do odbierania dźwięków przekazywanych przez powietrze, co jest typową drogą, jaką dźwięk dociera do ucha wewnętrznego.

Oznaczenia dla przewodnictwa powietrznego zazwyczaj różnią się w zależności od tego, czy mierzone są lewe czy prawe ucho:

Prawe Ucho: Zazwyczaj oznaczane jest przez symbol kółka (O). Kółka są umieszczane na audiogramie w punktach odpowiadających częstotliwości (mierzonej w hercach, Hz) i głośności (mierzonej w decybelach, dB) dźwięków, które pacjent jest w stanie usłyszeć.

Lewe Ucho: Zazwyczaj oznaczane jest przez symbol krzyżyka (x). Krzyżyki, podobnie jak kółka, są umieszczane na audiogramie w odpowiednich miejscach reprezentujących próg słyszenia dla różnych częstotliwości.

Podczas testu audiometrycznego, osoba badana nosi słuchawki, przez które przekazywane są dźwięki o różnych częstotliwościach i głośnościach. Audiolog zaznacza na audiogramie najcichsze dźwięki, które pacjent jest w stanie usłyszeć w każdej z testowanych częstotliwości, używając odpowiednich symboli dla każdego ucha. Wynikowy audiogram pokazuje wizualne przedstawienie słyszenia pacjenta, co jest kluczowe dla diagnozy i planowania leczenia niedosłuchu.

14. Co należy zrobić w przypadku nieprawidłowego wyniku przesiewowych badań słuchu u noworodka?

W przypadku nieprawidłowego wyniku przesiewowych badań słuchu u noworodka, ważne jest, aby podjąć odpowiednie kroki, aby dokładnie ocenić słuch dziecka i w razie potrzeby rozpocząć odpowiednie leczenie. Oto zalecane działania:

Powtórzenie Badania Przesiewowego: Często pierwszym krokiem jest ponowne przeprowadzenie badania przesiewowego. Nieprawidłowe wyniki mogą czasami wynikać z przejściowych problemów, takich jak obecność płynu w uchu środkowym, hałas w otoczeniu podczas testu lub niepokój dziecka.

Skierowanie do Specjalisty: Jeśli wyniki ponownego badania są nadal nieprawidłowe, dziecko powinno zostać skierowane do audiologa lub specjalisty w dziedzinie medycyny słuchu, aby przeprowadzić bardziej szczegółowe badania słuchu.

Dokładniejsze Badania Diagnostyczne: Do bardziej szczegółowych badań należą audiometria tonalna, tympanometria czy otoemisje akustyczne (OAE). W przypadku niemowląt często stosuje się również badanie potencjałów wywołanych pnia mózgu (ABR - Auditory Brainstem Response), które jest obiektywnym testem oceniającym działanie słuchu.

Planowanie Leczenia: Jeśli zostanie stwierdzony niedosłuch, ważne jest, aby jak najszybciej rozpocząć leczenie, które może obejmować aparaty słuchowe, terapię słuchową lub, w niektórych przypadkach, implant ślimakowy. Wczesna interwencja jest kluczowa dla rozwoju mowy i języka dziecka.

Monitoring i Wsparcie: Regularny monitoring słuchu i mowy dziecka jest ważny, aby zapewnić najlepsze możliwe wsparcie w jego rozwoju. Może to obejmować regularne wizyty u audiologa oraz specjalistów od wczesnej interwencji.

Wsparcie Rodziny: Rodzice i opiekunowie dziecka również mogą potrzebować wsparcia i edukacji na temat niedosłuchu i sposobów komunikacji z dzieckiem. Możliwe, że będą potrzebowali wsparcia psychologicznego oraz informacji o dostępnych zasobach i grupach wsparcia.

Wczesna diagnoza i interwencja są kluczowe dla zapewnienia, że dzieci z niedosłuchem osiągną swój pełny potencjał w komunikacji, rozwoju mowy i języka oraz ogólnym rozwoju.

15. Co oznaczają żółte i niebieskie certyfikaty w książeczce zdrowia dziecka w kontekście przesiewowych badań słuchu dzieci w Polsce?

W Polsce, w kontekście przesiewowych badań słuchu u noworodków i niemowląt, używa się specjalnych certyfikatów, które są wklejane do książeczki zdrowia dziecka. Te certyfikaty - żółty i niebieski - mają za zadanie dokumentować wyniki przesiewowych badań słuchu. Oto ich znaczenie:

Żółty Certyfikat: Zazwyczaj oznacza, że w przesiewowych badaniach słuchu (np. Otoemisyjne Badania Przesiewowe - OAE) wykryto potencjalny problem. W przypadku otrzymania żółtego certyfikatu zaleca się przeprowadzenie dalszych badań, aby dokładnie określić stan słuchu dziecka. To nie jest jeszcze diagnoza niedosłuchu, ale sygnał, że konieczne są dodatkowe badania.

Niebieski Certyfikat: Oznacza, że wyniki przesiewowych badań słuchu są prawidłowe. Dziecko przeszło testy przesiewowe bez wykrycia jakichkolwiek problemów ze słuchem. Otrzymanie niebieskiego certyfikatu jest więc dobrym znakiem i wskazuje, że słuch dziecka jest na tym etapie w normie.

Ważne jest, aby rodzice świadomie podchodzili do wyników badań przesiewowych. Żółty certyfikat nie oznacza jeszcze niedosłuchu, ale wskazuje na konieczność dalszej diagnostyki. Natomiast niebieski certyfikat, mimo że jest pozytywny, nie wyklucza konieczności obserwacji słuchu dziecka w przyszłości, zwłaszcza jeśli pojawiają się jakiekolwiek obawy dotyczące jego rozwoju słuchowego lub mowy.

16. **Jakie są przeciwwskazania do używania aparatów słuchowych na przewodnictwo powietrzne?**

Aparaty słuchowe na przewodnictwo powietrzne są powszechnie stosowanym rozwiązaniem w przypadku różnych form niedosłuchu, ale istnieją pewne sytuacje, w których ich używanie może nie być zalecane lub może być nieskuteczne. Oto niektóre z przeciwwskazań lub ograniczeń w stosowaniu tych aparatów:

Ekstremalny Niedosłuch: W przypadku bardzo głębokiego lub całkowitego niedosłuchu, aparaty słuchowe na przewodnictwo powietrzne mogą być niewystarczająco efektywne, ponieważ nie są w stanie dostarczyć wystarczającej amplifikacji dźwięku.

Chroniczne Infekcje Ucha: Osoby z chronicznymi infekcjami ucha środkowego, częstymi zapaleniami ucha, czy przewlekłym wyciekiem z ucha mogą mieć ograniczone możliwości używania tradycyjnych aparatów słuchowych, które wymagają bezpośredniego kontaktu z uchem zewnętrznym.

Alergie Skórne lub Problemy Dermatologiczne: Osoby z alergiami skórnymi, szczególnie w obszarze ucha, mogą mieć problemy z noszeniem aparatów słuchowych ze względu na drażniące działanie materiałów, z których są wykonane.

Anomalie Strukturalne Ucha: W przypadkach, gdy struktura ucha zewnętrznego lub środkowego jest nieprawidłowa (na przykład wrodzone malformacje), aparaty słuchowe na przewodnictwo powietrzne mogą nie pasować lub nie być skuteczne.

Brak Współpracy Pacjenta: W niektórych przypadkach, zwłaszcza u małych dzieci lub osób z określonymi problemami neurologicznymi, współpraca w zakresie noszenia i obsługi aparatów słuchowych może być ograniczona.

Problemy z Akumulacją Cerumenu: Nadmierna produkcja woskowiny w uchu może wpływać na efektywność i wygodę użytkowania aparatów słuchowych.

W przypadkach, gdy aparaty słuchowe na przewodnictwo powietrzne nie są odpowiednie, mogą być rozważane inne opcje, takie jak aparaty słuchowe na przewodnictwo kostne, implanty ślimakowe lub inne rodzaje wsparcia słuchowego. Ważne jest, aby każdy przypadek był indywidualnie oceniany przez specjalistę, który może zalecić najlepsze dostępne rozwiązanie.

17. **Jakie są najskuteczniejsze metody leczenia wysiękowego zapalenia ucha środkowego u dziecka?**

Wysiękowe zapalenie ucha środkowego (otitis media with effusion, OME) u dzieci jest powszechnym stanem, w którym płyn gromadzi się w uchu środkowym bez objawów infekcji. Leczenie OME zależy od różnych czynników, w tym wieku dziecka, nasilenia objawów, czasu trwania stanu oraz wpływu na słuch i rozwój mowy. Oto najskuteczniejsze metody leczenia tego stanu:

Obserwacja i Monitorowanie: W wielu przypadkach, zwłaszcza gdy objawy są łagodne i nie ma wpływu na słuch, zaleca się tzw. "watchful waiting" czyli obserwację i monitorowanie. Wysięk często ustępuje samoczynnie w ciągu kilku tygodni do miesięcy.

Leczenie Farmakologiczne: Antybiotyki nie są zazwyczaj zalecane w leczeniu OME, ponieważ nie ma aktywnej infekcji. W niektórych przypadkach mogą być stosowane leki przeciwhistaminowe lub kortykosteroidy, ale ich skuteczność w leczeniu OME jest ograniczona i mogą mieć skutki uboczne.

Rurki Wentylacyjne (Dreny): W przypadkach przewlekłego OME, szczególnie gdy towarzyszy mu utrata słuchu lub wpływa na rozwój mowy dziecka, lekarz może zalecić umieszczenie rurek wentylacyjnych (drenów) w błonie bębenkowej. To małe, cylindryczne urządzenie pozwala na odprowadzenie płynu z ucha środkowego i wyrównanie ciśnienia powietrza.

Usuwanie Migdałków Podniebiennych i Trzeciego Migdałka (Adenotomia): W niektórych przypadkach, zwłaszcza gdy powiększone migdałki przyczyniają się do problemów z wentylacją ucha środkowego, może być zalecone usunięcie migdałków podniebiennych (tonsillektomia) lub trzeciego migdałka (adenotomia).

Terapia Mowy: Jeśli wysiękowe zapalenie ucha środkowego wpływa na rozwój mowy dziecka, może być wskazana terapia mowy.

Zachowanie Dobrej Higieny Nosowej: Regularne czyszczenie nosa i stosowanie soli fizjologicznej mogą pomóc w utrzymaniu drożności dróg oddechowych i poprawić wentylację ucha środkowego.

Unikanie Czynników Ryzyka: Takie działania jak unikanie dymu tytoniowego i alergenów mogą pomóc zmniejszyć ryzyko wystąpienia lub nasilenia OME.

Ważne jest, aby leczenie było dostosowane indywidualnie do potrzeb dziecka i odbywało się pod nadzorem lekarza specjalisty. W niektórych przypadkach, szczególnie gdy wysięk utrzymuje się przez dłuższy czas lub powoduje problemy ze słuchem, interwencja medyczna może być niezbędna.

18. Jak wygląda wynik badania audiometrii słownej i jak go interpretować?

Audiometria słowna to badanie, które ocenia, jak dobrze osoba rozumie mowę i jest w stanie rozróżnić słowa. Wynik tego badania jest zazwyczaj przedstawiony w postaci procentu poprawnie rozpoznanych słów. Oto, jak wygląda wynik badania i jak go interpretować:

Wynik Procentowy: Wynik audiometrii słownej jest zwykle przedstawiany jako procent poprawnie rozpoznanych słów. Na przykład, jeśli pacjent poprawnie rozpoznał 80% ze słyszanych słów, wynik to 80%.

Poziom Prezentacji: Słowa są prezentowane na różnych poziomach głośności, zwykle mierzone w decybelach (dB). Wynik jest często podawany z określeniem poziomu głośności, przy którym słowa były prezentowane. Na przykład, wynik "80% przy 50 dB" oznacza, że pacjent poprawnie rozpoznał 80% słów, które były mu prezentowane przy głośności 50 dB.

Interpretacja:

Normalny Wynik: Ogólnie rzecz biorąc, wyniki w zakresie 90-100% są uważane za normalne.

Lekkie Problemy z Rozumieniem Mowy: Wyniki w zakresie 75-89% mogą wskazywać na lekkie problemy z rozumieniem mowy.

Umiarkowane do Znaczących Trudności: Wyniki poniżej 75% często wskazują na umiarkowane do znaczących trudności w rozumieniu mowy.

Poziom Głośności: Jeśli pacjent osiąga dobre wyniki, ale tylko przy wyższych poziomach głośności, może to wskazywać na obecność niedosłuchu.

Porównanie z Audiometrią Tonową: Wyniki audiometrii słownej są często porównywane z wynikami audiometrii tonowej (która mierzy progowe poziomy słyszenia dla różnych częstotliwości dźwięku). Rozbieżności między tymi dwoma badaniami mogą dostarczać ważnych informacji diagnostycznych.

Znaczenie dla Rehabilitacji: Wyniki audiometrii słownej są bardzo ważne przy planowaniu rehabilitacji słuchowej, w tym doborze i kalibracji aparatów słuchowych lub planowania terapii logopedycznej.

Pamiętaj, że interpretacja wyników audiometrii słownej powinna być przeprowadzona przez wykwalifikowanego audiologa lub lekarza specjalistę, który weźmie pod uwagę całościowy obraz stanu słuchu pacjenta, a także inne czynniki, takie jak wiek, ogólny stan zdrowia, i historię medyczną.

19. Jakie są wyniki otoskopii w przypadku otosklerozy?

Otoskleroza to stan chorobowy, w którym dochodzi do nieprawidłowego wzrostu kości w uchu środkowym, co może prowadzić do postępującego niedosłuchu przewodzeniowego. Otoskopia, czyli badanie ucha za pomocą otoskopu, jest jednym z podstawowych narzędzi diagnostycznych stosowanych przez lekarzy, choć nie zawsze może wykryć bezpośrednie oznaki otosklerozy. Oto, co może być widoczne podczas otoskopii w przypadku tej choroby:

Normalny Wygląd Błony Bębenkowej: W większości przypadków otosklerozy, błona bębenkowa wygląda normalnie, bez znaków zapalenia czy infekcji. Nie ma typowych zmian, które można by bezpośrednio skojarzyć z otosklerozą.

Znak Schwartze'a: W niektórych przypadkach, można zaobserwować tzw. znak Schwartze'a, który polega na prześwitywaniu przez błonę bębenkową hiperemii (zwiększonego ukrwienia) kości kowadełka. Jest to jednak znak rzadko występujący.

Brak Widocznych Oznak Zapalenia: Podczas otoskopii nie są zwykle widoczne oznaki ostrego zapalenia ucha, takie jak zaczerwienienie, obrzęk, czy obecność płynu w uchu środkowym.

Ponieważ otoskleroza dotyczy głównie struktur kostnych w uchu wewnętrznym i środkowym, które nie są bezpośrednio widoczne podczas otoskopii, diagnoza zwykle opiera się na kombinacji historii medycznej, wyników testów słuchu (audiometria tonalna i impedancyjna) oraz, w niektórych przypadkach, badań obrazowych, takich jak tomografia komputerowa ucha.

Otoskleroza jest zazwyczaj diagnozowana u osób w młodym wieku dorosłym i częściej występuje u kobiet. Wczesna diagnoza i odpowiednie leczenie, które może obejmować zarówno terapię farmakologiczną (np. fluorowce), jak i chirurgiczne (stapedektomia lub stapedotomia), są kluczowe dla zarządzania stanem i minimalizowania wpływu na słuch.

20. Jakie wszczepialne protezy słuchu można stosować u dzieci?

Wszczepialne protezy słuchu są ważną opcją dla dzieci z ciężkim niedosłuchem lub głuchotą, u których tradycyjne aparaty słuchowe nie są wystarczająco skuteczne. Istnieje kilka rodzajów wszczepialnych protez słuchu, które mogą być stosowane u dzieci:

Implanty Ślimakowe (Cochlear Implants): Są to urządzenia, które omijają uszkodzone części ucha wewnętrznego i bezpośrednio stymulują nerw słuchowy. Są zalecane dla dzieci z głębokim niedosłuchem lub głuchotą obustronną. Implant ślimakowy składa się z zewnętrznego procesora dźwięku, który przetwarza dźwięki, oraz wewnętrznego implantu, który przekazuje sygnały elektryczne do nerwu słuchowego. Implantacja jest możliwa nawet u bardzo małych dzieci.

Implanty Kostne (Bone Anchored Hearing Aids, BAHA): Są to urządzenia, które przekazują dźwięk bezpośrednio do wewnętrznego ucha poprzez kość czaszkową. Są one opcją dla dzieci z niedosłuchem przewodzeniowym, mieszanym lub jednostronnym, w przypadkach, gdy tradycyjne aparaty słuchowe nie są odpowiednie (np. z powodu wad anatomicznych ucha zewnętrznego lub środkowego). BAHA wymaga implantacji chirurgicznej, ale jest mniej inwazyjna niż implant ślimakowy.

Implanty Środkowouczne (Middle Ear Implants): To alternatywa dla tradycyjnych aparatów słuchowych, gdzie urządzenie jest wszczepiane w uchu środkowym. Są one zazwyczaj zalecane dla dzieci z umiarkowanym do ciężkiego niedosłuchu sensorycznego. Te urządzenia wymagają operacji, ale mogą oferować lepszą jakość dźwięku w porównaniu do zewnętrznych aparatów słuchowych.

Implanty Pniowe (Auditory Brainstem Implants, ABI): Są to urządzenia stosowane głównie u dzieci z neurofibromatozą typu 2 lub innymi schorzeniami, które uniemożliwiają stosowanie implantów ślimakowych. ABI jest implantowane bezpośrednio w pień mózgu, omijając ślimak i nerw słuchowy.

Wybór odpowiedniego rodzaju wszczepialnej protezy słuchu zależy od indywidualnej sytuacji dziecka, w tym od rodzaju i stopnia niedosłuchu, stanu anatomicznego ucha, wieku dziecka, a także od ogólnego stanu zdrowia i rozwoju. Decyzja o wszczepieniu protezy słuchowej powinna być podjęta po konsultacji z zespołem specjalistów, w tym audiologami, otolaryngologami, a także logopedami i pedagogami specjalnymi.

Ważne jest, aby pamiętać, że wszczepienie takich urządzeń to tylko część procesu. Po wszczepieniu, dziecko wymaga ciągłej terapii logopedycznej i rehabilitacji słuchowej, aby nauczyć się interpretować dźwięki, które odbiera, co jest kluczowe dla rozwoju mowy i języka. Wsparcie edukacyjne i społeczne również odgrywa znaczącą rolę w integracji dziecka z niedosłuchem w środowisku szkolnym i społecznym.

Questions to ChatGPT in English language version

1. What is the CROS system?
2. What is conductive hearing loss?
3. What frequencies are responsible for the reception of speech sounds in children?
4. What can be the causes of otitis in a child?
5. How to interpret the test of tonal audiometry, what is the norm of hearing?
6. What is the ABR test in a child?
7. What is newborn hearing screening?
8. What is the course of exudative otitis media in a child?
9. How can hearing be tested in a one-year-old child?
10. What is the verbal audiometry test?
11. What types of tympanograms are distinguished in impedance audiometry?
12. For what purpose is an ear impression performed in children?
13. How to determine air conduction in the audiogram?
14. What should be done in the case of an abnormal result of hearing screening in a newborn?
15. What do the yellow and blue certificates in the child's health book mean in the context of hearing screening of children in Poland?
16. What are the contraindications to the use of air-conduction hearing aids?
17. What are the most effective methods of treatment of exudative otitis media in a child?
18. What is the result of a verbal audiometry test and how to interpret it?
19. What are the results of otoscopy for otosclerosis?
20. What implantable hearing prostheses can be used in children?

Questions with ChatGPT answers in English language version

1. **What is the CROS system?**

The CROS Hearing Aid System is a solution designed to help individuals with unilateral hearing loss. Unilateral hearing loss means that a person has significant hearing loss in one ear while the other ear has normal or near-normal hearing. The CROS system is particularly useful for those who have no usable hearing in one ear.

"CROS" stands for Contralateral Routing of Signals. Here's how the CROS Hearing Aid System typically works:

Microphone on the Deaf Side: A microphone is placed on the side of the head with hearing loss. This microphone picks up sounds from the deaf or impaired ear.

Transmission to the Better Ear: The captured sound is wirelessly transmitted to a hearing aid or receiver located on the better-hearing side. This allows the individual to hear sounds from both sides.

Improved Awareness: By transmitting sounds from the impaired side to the better-hearing ear, the CROS system helps individuals with unilateral hearing loss to be more aware of their surrounding environment and to better understand the directionality of sounds.

This technology is different from traditional hearing aids, which are designed to amplify sounds for individuals with hearing loss in both ears. The CROS Hearing Aid System is specifically tailored for those with one-sided hearing loss, providing a more comprehensive listening experience.

2. **What is conductive hearing loss?**

Conductive hearing loss is a type of hearing impairment that occurs when there is a problem with the transmission of sound waves from the outer or middle ear to the inner ear. This type of hearing loss can result from various issues that affect the ear's ability to conduct sound effectively.

Common causes of conductive hearing loss include:

Ear Infections: Infections in the ear canal or middle ear can cause inflammation and fluid buildup, affecting the movement of the eardrum and the small bones (ossicles) in the middle ear.

Blockage in the Ear Canal: Earwax (cerumen) buildup or the presence of a foreign object in the ear can block sound waves from reaching the eardrum.

Perforated Eardrum: A tear or hole in the eardrum can disrupt the normal transmission of sound.

Otosclerosis: Abnormal bone growth in the middle ear can interfere with the movement of the stapes bone, one of the three ossicles, leading to conductive hearing loss.

Malformation of the Ear: Some individuals may be born with structural abnormalities in the outer or middle ear that result in conductive hearing loss.

Conductive hearing loss is generally treatable, and in many cases, medical intervention or surgical procedures can help restore or improve hearing. Treatment options may include the removal of earwax, antibiotic treatment for infections, surgical repair of a perforated eardrum, or procedures to correct abnormalities in the middle ear.

It's important to note that conductive hearing loss is distinct from sensorineural hearing loss, which involves damage to the inner ear or auditory nerve and is often irreversible. In some cases, individuals may experience a combination of conductive and sensorineural hearing loss, known as mixed hearing loss. If you suspect hearing loss, it's advisable to consult with an audiologist or healthcare professional for a comprehensive hearing evaluation.

3. What frequencies are responsible for the reception of speech sounds in children?

Speech sounds cover a range of frequencies, and different frequencies contribute to the perception of various speech sounds. In general, the frequencies associated with speech sounds in children (as well as in adults) fall within the range of human hearing, which is approximately 20 Hz to 20,000 Hz. However, the key frequencies for speech understanding are typically found in the mid-frequency range.

The fundamental frequency (or pitch) of human speech, which is critical for conveying the intonation and emotional aspects of speech, usually falls between 125 Hz and 250 Hz. Vowels in speech are characterized by their formant frequencies, with the first formant typically ranging from around 300 Hz to 700 Hz and the second formant ranging from around 800 Hz to 1200 Hz.

Consonants, on the other hand, span a wider range of frequencies. Some of the higher-frequency components of speech associated with consonant sounds can extend beyond 2,000 Hz.

It's important to note that speech perception involves the integration of information across various frequencies, and the specific frequencies that contribute to the understanding of speech sounds can vary depending on the linguistic and acoustic characteristics of the language being spoken.

In the context of assessing and managing hearing loss in children, audiologists often focus on the frequency range associated with speech sounds to ensure that amplification devices (such as hearing aids) provide sufficient amplification in these critical frequency regions for effective speech comprehension and language development.

4. What can be the causes of otitis in a child?

Otitis, which refers to inflammation of the ear, can manifest in different forms. The two main types of otitis in children are otitis media and otitis externa, each with its own set of causes:

Otitis Media:

Bacterial or Viral Infection: Otitis media is often associated with infections caused by bacteria or viruses. Respiratory infections, such as the common cold, can lead to the accumulation of fluid in the middle ear, creating an environment conducive to bacterial or viral growth.

Eustachian Tube Dysfunction: The Eustachian tubes, which connect the middle ear to the back of the throat, help regulate air pressure in the ear. Dysfunction of these tubes, often due to anatomical factors or inflammation, can lead to fluid buildup and otitis media.

Allergies: Allergic reactions may contribute to inflammation and congestion in the Eustachian tubes, increasing the risk of otitis media.

Environmental Factors: Exposure to secondhand smoke or pollutants can be a risk factor for ear infections in children.

Otitis Externa (Swimmer's Ear):

Moisture and Humidity: Otitis externa is commonly associated with excess moisture in the ear canal, which creates an environment conducive to bacterial or fungal growth. This can be more prevalent in warm and humid conditions.

Trauma or Irritation: Scratching the ear canal with objects like cotton swabs or inserting foreign objects can damage the delicate skin of the ear canal, leading to inflammation.

Excessive Earwax: Buildup of earwax can create a favorable environment for bacterial growth and contribute to otitis externa.

Preventive measures for both types of otitis in children include practicing good hygiene, avoiding exposure to smoke, and addressing allergies when present. Prompt treatment of respiratory infections and avoiding prolonged exposure to water in the ear can help prevent ear infections.

If a child exhibits symptoms of ear infection, such as ear pain, drainage, or changes in hearing, it's important to consult a healthcare professional for proper diagnosis and treatment. Treatment may involve antibiotics for bacterial infections, pain management, and, in some cases, the placement of ear tubes for recurrent otitis media.

5. How to interpret the test of tonal audiometry, what is the norm of hearing?

Tonal audiometry is a hearing test that measures a person's ability to hear sounds at different frequencies and intensities. The results of tonal audiometry are typically presented on an audiogram, which is a graph that shows the individual's hearing thresholds at various frequencies. Here's how to interpret tonal audiometry results:

Audiogram Basics:

The horizontal axis of the audiogram represents frequencies, usually ranging from low to high frequencies (measured in Hertz, Hz).

The vertical axis represents intensity or loudness, measured in decibels (dB). The top of the audiogram represents loud sounds, and the bottom represents soft sounds.

Symbols on the Audiogram:

O (circle): Represents the right ear.

X (cross): Represents the left ear.

Audiometric Thresholds: The points on the graph where the symbols are plotted indicate the softest sounds that the individual can hear at each frequency. The lower the threshold, the better the hearing.

Degrees of Hearing Loss:

Normal Hearing: Thresholds at or below 20 dB across all frequencies are considered within the normal range.

Mild Hearing Loss: Thresholds between 20 and 40 dB.

Moderate Hearing Loss: Thresholds between 40 and 60 dB.

Severe Hearing Loss: Thresholds between 60 and 80 dB.

Profound Hearing Loss: Thresholds above 80 dB.

Configuration of Hearing Loss:

The shape or pattern of the audiogram can provide information about the configuration of hearing loss (e.g., flat, sloping, rising).

Speech Frequencies:

Important speech frequencies are typically tested at 500 Hz, 1000 Hz, 2000 Hz, 4000 Hz, and 8000 Hz.

It's important to note that the interpretation of tonal audiometry results should be done by a qualified audiologist or hearing healthcare professional. They will consider the entire audiogram, assess both ears, and provide appropriate recommendations based on the individual's hearing profile.

If you have a specific audiogram result and would like more detailed information, consulting with an audiologist is recommended, as they can provide personalized guidance based on the specific context and needs of the individual.

6. What is the ABR test in a child?

The Auditory Brainstem Response (ABR) test, also known as brainstem auditory evoked response (BAER), is a diagnostic test used to evaluate the auditory nerve and brainstem's responses to sound. It is commonly employed in both adults and children, including infants and newborns. In children, the ABR test is particularly useful when traditional behavioral hearing tests are challenging to conduct due to age or developmental factors.

Here's how the ABR test works:

Electrode Placement: Small electrodes are placed on the child's scalp and sometimes on the earlobes. These electrodes pick up electrical responses generated by the auditory nerve and brainstem in response to sound stimuli.

Stimuli Presentation: The child is exposed to a series of brief clicking sounds or tone bursts through earphones. These sounds are presented at different intensities and frequencies.

Measurement of Responses: The electrodes pick up the electrical responses generated by the auditory nerve and brainstem as a result of the presented sounds. The responses are then recorded and analyzed.

Threshold Determination: The ABR test can provide information about the child's hearing thresholds, which are the softest sounds they can detect.

The ABR test is valuable in several scenarios, including:

Newborn Hearing Screening: It is often used as part of newborn hearing screening programs to identify hearing issues early in infancy.

Young Children: When traditional behavioral testing is challenging due to a child's age or developmental stage.

Neurological Assessment: It can provide information about the integrity of the auditory nerve and brainstem pathways.

The ABR test is a non-invasive and objective measure of hearing function. While it does not provide information about the child's perception of speech or language development, it is an essential tool in the comprehensive evaluation of a child's auditory system. The results of the ABR test can help guide further diagnostic and intervention efforts if hearing issues are identified. A qualified audiologist typically administers and interprets the ABR test.

7. What is newborn hearing screening?

Newborn hearing screening is a set of tests designed to identify hearing issues in infants shortly after birth. The goal of newborn hearing screening is to identify hearing loss early so that intervention and treatment can begin promptly, promoting optimal language and speech development. Early detection is crucial because infants learn language and communication skills during their early months and years of life, and hearing is fundamental to this process.

There are two main types of newborn hearing screening tests:

Otoacoustic Emissions (OAE) Test:

In this test, a small probe is placed in the baby's ear, and it emits a sound. If the baby's inner ear (cochlea) is functioning properly, it will produce an echo-like response called an otoacoustic emission. The presence of these emissions suggests normal hearing.

Auditory Brainstem Response (ABR) Test:

This test measures the electrical activity of the auditory nerve and brainstem in response to sound. Small electrodes are placed on the baby's scalp, and the baby listens to clicking sounds. The test records the electrical responses, and the audiologist analyzes them to determine the baby's hearing status.

Newborn hearing screening is usually conducted in the hospital shortly after birth, often before the baby is discharged. The tests are quick, non-invasive, and typically do not cause discomfort to the infant. If the initial screening suggests a potential hearing issue, further diagnostic evaluations, such as a comprehensive audiological assessment, may be recommended.

It's important to note that not all babies who fail the initial screening have permanent hearing loss. Some infants may have temporary hearing issues due to factors like fluid in the ear or debris, and a follow-up evaluation helps differentiate between temporary and permanent hearing loss.

Early intervention and appropriate support for infants identified with hearing loss can significantly impact their language and communication development. If you have concerns about your baby's hearing, it's important to discuss them with healthcare providers, and if necessary, follow through with recommended hearing screening and evaluations.

8. What is the course of exudative otitis media in a child?

Exudative otitis media (EOM), also known as serous or secretory otitis media, is a condition characterized by the accumulation of fluid in the middle ear without signs of acute infection. This condition is commonly seen in children and can have various courses, depending on factors such as the underlying cause, treatment, and individual response. Here is a general overview of the course of exudative otitis media in a child:

Onset and Development:

EOM often develops as a result of dysfunction of the Eustachian tube, which connects the middle ear to the back of the throat. This dysfunction can lead to poor ventilation and drainage of the middle ear, causing fluid accumulation.

Asymptomatic or Mild Symptoms:

In some cases, children with EOM may not exhibit noticeable symptoms, or they may experience only mild symptoms such as a feeling of fullness in the ear, mild hearing loss, or occasional popping sounds.

Persistence of Fluid:

The fluid in the middle ear may persist for an extended period, leading to a chronic or recurrent condition. This persistence can be influenced by factors like allergies, exposure to secondhand smoke, or anatomical issues with the Eustachian tube.

Hearing Loss:

One of the primary concerns with EOM is its impact on hearing. The accumulation of fluid in the middle ear can interfere with the transmission of sound, resulting in conductive hearing loss.

Spontaneous Resolution:

In some cases, EOM may resolve spontaneously without intervention. As a child grows and the Eustachian tube matures, the ability to maintain proper ventilation and drainage of the middle ear may improve, leading to resolution of the condition.

Treatment Options:

When EOM persists or causes significant hearing loss, healthcare providers may consider interventions. Treatment options may include the management of underlying factors (such as allergies), placement of ear tubes (tympanostomy tubes) to allow fluid drainage, or other medical interventions.

Follow-up and Monitoring:

Children with EOM typically undergo regular follow-up evaluations to monitor the condition and assess hearing. Healthcare providers may adjust the treatment plan based on the child's progress.

It's important for parents and caregivers to work closely with healthcare providers to manage exudative otitis media effectively. Regular monitoring and appropriate interventions can help minimize the impact of EOM on a child's hearing and overall well-being.

9. How can hearing be tested in a one-year-old child?

Testing the hearing of a one-year-old child requires specialized techniques designed to accommodate their age and developmental stage. Here are two common methods used to assess hearing in infants and toddlers, including one-year-olds:

Behavioral Observation Audiometry (BOA):

This method involves observing the child's behavioral responses to sound. While the child is engaged in activities or play, the audiologist or healthcare professional presents sounds at different frequencies and volumes and monitors the child's reactions.

Responses may include the child turning their head toward the sound, displaying a startle reflex, or showing heightened attention. This method is suitable for children as young as a few months old.

Visual Reinforcement Audiometry (VRA):

VRA is a more structured method that involves pairing sound stimuli with a visual reward. The child is conditioned to associate a sound with a rewarding visual stimulus, such as a lighted toy or an animated display. When the child responds to the sound, they are rewarded visually.

This method is often used with children between six months and two years of age. The child is typically seated on a caregiver's lap, and speakers are used to present sounds from different directions.

It's important to note that testing the hearing of a one-year-old may require adaptation and flexibility based on the child's behavior and attention span. The testing environment is often designed to be child-friendly and engaging to encourage the child's participation.

If hearing concerns are identified during these initial screenings, more comprehensive testing, such as auditory brainstem response (ABR) testing, may be recommended. ABR is a neurophysiological test that measures the electrical activity of the auditory nerve and brainstem in response to sound.

Parents and caregivers play a crucial role in providing information about their child's responses to sound and should communicate any concerns to healthcare professionals. If there are suspected hearing issues or if the child is at risk for hearing loss, early intervention and further diagnostic testing may be recommended to address any potential hearing-related challenges.

10. What is the verbal audiometry test?

Verbal audiometry is a type of hearing test that assesses an individual's ability to understand and repeat spoken words or phrases. This test is commonly used to evaluate the functional aspects of hearing, particularly in terms of speech perception and discrimination. Verbal audiometry is often administered to individuals who are old enough to respond verbally, typically starting around age 5 or 6.

Here's an overview of how verbal audiometry is typically conducted:

Word Recognition Testing:

During a word recognition test, the individual listens to a list of single-syllable words or two-syllable words presented at a comfortable listening level. The words are usually spoken by the audiologist through headphones.

Scoring Responses:

The individual is then asked to repeat each word they hear. The audiologist records the individual's responses, noting which words were repeated correctly and which were misunderstood or not heard accurately.

Percentage of Correct Responses:

The results are expressed as a percentage of correct responses. This percentage reflects the individual's ability to accurately recognize and repeat the presented words.

Speech Discrimination:

In addition to word recognition testing, speech discrimination testing may be performed. This involves presenting sentences or phrases, and the individual is asked to repeat them. This type of testing provides information about the individual's ability to understand speech in a more complex context.

Verbal audiometry is an important component of a comprehensive audiological assessment, as it assesses how well an individual can understand and communicate with spoken language. The results of verbal audiometry, along with other tests like pure-tone audiometry and tympanometry, contribute to the overall understanding of a person's hearing abilities and may guide recommendations for intervention, such as hearing aids or other assistive devices.

It's important to note that verbal audiometry is typically used with individuals who have sufficient language and cognitive skills to participate in the test. For younger children or individuals with developmental or communication challenges, alternative methods, such as play audiometry or visual reinforcement audiometry, may be employed to assess hearing abilities.

11. What types of tympanograms are distinguished in impedance audiometry?

Tympanometry is a test used in impedance audiometry to assess the mobility of the eardrum (tympanic membrane) and the middle ear system. The test measures the compliance of the middle ear as air pressure in the ear canal is varied. Tympanograms are graphical representations of the results, and they help audiologists diagnose various middle ear conditions. There are several types of tympanograms, and they are categorized based on their shape and characteristics:

Type A Tympanogram:

This is considered a normal tympanogram. It shows a peak in compliance at the midpoint of the pressure range tested. The peak indicates that the eardrum and middle ear system are functioning well, and there is normal movement in response to changes in air pressure.

Type As Tympanogram:

A Type As tympanogram has a reduced peak compliance, suggesting reduced mobility of the tympanic membrane. This pattern is often associated with stiffening of the middle ear system, which can occur in conditions such as otosclerosis or partial ossicular discontinuity.

Type Ad Tympanogram:

A Type Ad tympanogram also has reduced peak compliance, but with an abnormally deep ear canal volume. This pattern is often associated with a flaccid or hypermobile tympanic membrane, which can be seen in conditions like a patulous Eustachian tube.

Type B Tympanogram:

A Type B tympanogram does not show a clear peak and is flat across the pressure range. This pattern can indicate a lack of mobility in the eardrum, often seen in conditions such as middle ear effusion (fluid in the middle ear) or a tympanic membrane perforation.

Type C Tympanogram:

A Type C tympanogram has a peak compliance shifted to the negative pressure side of the graph, indicating negative middle ear pressure. This pattern is often associated with Eustachian tube dysfunction or congestion, where the pressure in the middle ear is lower than normal.

These tympanogram types provide valuable information about the status of the middle ear, Eustachian tube function, and potential issues affecting hearing. Tympanometry is often performed as part of a comprehensive audiological evaluation to assess the overall health of the middle ear and contribute to the diagnosis and management of various ear conditions.

12. **For what purpose is an ear impression performed in children?**

Ear impressions, also known as earmolds or ear impressions, involve making a mold or impression of the ear's external shape and ear canal. These impressions are custom-made for an individual and are used for various purposes. In the context of children, ear impressions may be performed for the following purposes:

Hearing Aid Fittings:

Ear impressions are commonly used in the fitting of custom hearing aids for children. Custom hearing aids are designed to fit the unique shape and size of the child's ear, providing a comfortable and secure fit. The impressions allow the hearing healthcare professional to create hearing aids tailored to the child's specific ear anatomy.

Earmold for Hearing Protection:

Children may require hearing protection in certain noisy environments, such as concerts, sporting events, or loud recreational activities. Custom earmolds can be created based on ear impressions to provide a snug and effective fit, offering personalized hearing protection.

In-Ear Monitors for Musicians:

If a child is involved in music, particularly live performances, custom in-ear monitors can be created based on ear impressions. These monitors provide a comfortable and secure fit, delivering high-quality sound while also protecting the ears from excessive noise.

Swim Plugs or Water Protection:

Some children may benefit from custom swim plugs or water protection earmolds. These are designed to keep water out of the ears during activities such as swimming or bathing. The ear impressions help create a precise fit to prevent water from entering the ear canal.

Communication Earpieces:

In certain situations, such as for children with hearing loss who use communication devices, custom-molded earpieces may be needed. These earpieces can be created based on ear impressions to ensure a comfortable and effective fit for communication devices.

It's important to note that making ear impressions for children requires a gentle and child-friendly approach. Professionals, such as audiologists or hearing healthcare providers, with experience working with children, can ensure that the process is comfortable and stress-free for the child.

Overall, ear impressions for children serve a variety of purposes, all aimed at improving their auditory experiences, protecting their hearing, and enhancing their overall quality of life in various environments.

13. **How to determine air conduction in the audiogram?**

Air conduction testing in an audiogram is used to assess an individual's ability to hear sounds transmitted through the air. The audiogram is a graph that shows the softest sounds a person can hear at different frequencies (pitch) and intensities (volume). Air conduction testing involves the use of headphones or inserts, and the individual responds to various tones presented by the audiologist. Here's how air conduction testing is typically conducted:

Audiometric Equipment:

The audiologist uses specialized audiometric equipment, including calibrated headphones or inserts and an audiometer. The audiometer generates pure tones at different frequencies and intensities.

Frequency Selection:

The audiologist selects specific frequencies to be tested. Standard audiometric frequencies include 250 Hz, 500 Hz, 1000 Hz, 2000 Hz, 4000 Hz, and 8000 Hz. These frequencies are measured in Hertz (Hz).

Intensity Levels:

For each selected frequency, the audiologist presents tones at different intensity levels, measured in decibels hearing level (dB HL). The intensity starts at a level that is typically easy to hear, and then decreases in 5 or 10 dB steps until the softest sound that the individual can hear is determined.

Patient Response:

The individual being tested responds to each tone by pressing a button, raising a hand, or providing another predetermined signal when they hear the sound. The responses are recorded by the audiologist.

Plotting the Audiogram:

The results are plotted on an audiogram, with frequency on the horizontal axis and intensity (dB HL) on the vertical axis. The symbols on the audiogram represent the softest sounds the individual can hear at each tested frequency.

Threshold Determination:

The softest intensity level at which the individual can hear each frequency is known as the hearing threshold. The hearing thresholds are used to assess the degree of hearing loss, if any, at different frequencies.

Air conduction testing assesses the entire auditory system, from the outer ear to the inner ear and beyond. It provides information about the individual's overall hearing sensitivity and helps identify the type and configuration of hearing loss. If a hearing loss is identified, further testing, such as bone conduction testing, may be conducted to determine the specific site of the hearing impairment within the auditory system.

14. What should be done in the case of an abnormal result of hearing screening in a newborn?

If a newborn's hearing screening results are abnormal, it is important to follow up with additional testing and evaluation promptly. Early detection of hearing loss is crucial for intervention and support to ensure optimal language and communication development. Here are the general steps to be taken when an abnormal result is obtained in a newborn hearing screening:

Repeat Screening:

In some cases, a false positive result may occur due to factors such as debris in the ear canal or the infant being unsettled during the initial screening. Before proceeding with additional testing, it is common to repeat the hearing screening to confirm the result.

Diagnostic Auditory Brainstem Response (ABR) Testing:

If the repeat screening still indicates a potential hearing issue, the next step is often a diagnostic Auditory Brainstem Response (ABR) test. ABR is a more comprehensive and specific test that measures the electrical activity of the auditory nerve and brainstem in response to sound. It provides information about the presence, degree, and type of hearing loss.

Medical Evaluation:

If the ABR test confirms hearing loss, the infant should undergo a medical evaluation by an otolaryngologist (ear, nose, and throat specialist) or a pediatric audiologist. The medical evaluation helps identify any underlying medical conditions or anatomical issues that may be contributing to the hearing loss.

Intervention Planning:

Once the type and degree of hearing loss are determined, an intervention plan can be developed. This plan may include recommendations for hearing aids, cochlear implants (if appropriate), and early intervention services to support the child's communication and language development.

Family Support and Education:

Supporting the family is an essential aspect of managing hearing loss in a newborn. Families should receive information and resources to help them understand the implications of hearing loss and how to support their child's communication and development. Educational programs and resources for families with children who are deaf or hard of hearing are often available.

Follow-up Monitoring:

Regular follow-up appointments and monitoring are crucial to assess the child's progress and make any necessary adjustments to the intervention plan. Ongoing audiological evaluations help track changes in hearing and ensure that appropriate interventions are in place as the child grows.

It's important for parents and caregivers to actively participate in the process, communicate openly with healthcare professionals, and seek support from relevant services and organizations specializing in early intervention for hearing loss. Early identification and intervention significantly improve outcomes for children with hearing loss.

15. **What do the yellow and blue certificates in the child's health book mean in the context of hearing screening of children in Poland?**

As of my last knowledge update in January 2022, I do not have specific information about the color-coding system used in child health books in Poland for hearing screening certificates. Health systems and administrative procedures can evolve, and it's possible that there have been changes or updates since then.

In many countries, including Poland, health books or child health records often contain information related to the child's health, vaccinations, growth, and development milestones. Hearing screening is an essential component of early childhood health monitoring, and certificates may be issued to document the results of hearing screening tests.

To obtain accurate and up-to-date information about the specific color-coding system used for hearing screening certificates in Poland, I recommend checking with local health authorities, pediatricians, or the relevant government health agencies. They can provide details about the current practices, protocols, and

the meaning of different colors or codes on hearing screening certificates in the context of child health records in Poland.

16. What are the contraindications to the use of air-conduction hearing aids?

Air-conduction hearing aids are commonly used to amplify sounds and improve hearing for individuals with hearing loss. While they are generally safe and effective, there are specific situations or conditions where the use of air-conduction hearing aids may not be advisable. Contraindications to the use of air-conduction hearing aids include:

Presence of Acute Ear Infections:

Individuals with active or acute ear infections, such as otitis media with drainage, may be advised to avoid using hearing aids until the infection is treated and resolved. Introducing hearing aids in the presence of an active infection can potentially worsen the condition.

Severe External Otitis or Dermatitis:

Severe external otitis (inflammation of the ear canal) or dermatitis of the ear may be a contraindication to using traditional air-conduction hearing aids. The presence of inflammation or open sores may make wearing hearing aids uncomfortable or exacerbate the existing condition.

Drainage or Otitis Externa with Inflammation:

Conditions involving persistent drainage or inflammation of the ear canal, such as chronic otitis externa, may be contraindications to using air-conduction hearing aids. These conditions may interfere with the proper functioning and fit of hearing aids.

Anatomical Abnormalities or Deformities:

Some individuals may have anatomical abnormalities or deformities of the ear that make it challenging to achieve a proper fit with conventional hearing aids. In such cases, alternative hearing solutions may be explored.

Allergic Reactions:

Allergic reactions to materials used in hearing aids (e.g., the casing, ear molds, or adhesives) may be a contraindication. In these cases, individuals may need hypoallergenic materials or different hearing aid options.

Inability to Tolerate Hearing Aid Use:

Some individuals may experience discomfort, irritation, or a sense of being overwhelmed when using hearing aids. In such cases, it's important to address the specific issues causing discomfort and explore alternative solutions or adjustments.

It's crucial for individuals with hearing loss to undergo a comprehensive hearing evaluation by an audiologist or hearing healthcare professional. The audiologist can assess the individual's specific needs, determine the type and degree of hearing loss, and recommend suitable hearing solutions based on the individual's overall health and anatomical considerations.

Ultimately, the decision to use air-conduction hearing aids or explore alternative solutions depends on the individual's unique circumstances and the guidance of qualified healthcare professionals.

17. What are the most effective methods of treatment of exudative otitis media in a child?

Exudative otitis media (EOM), also known as serous or secretory otitis media, is a condition characterized by the accumulation of fluid in the middle ear without signs of acute infection. The management of EOM in

children often depends on the underlying cause and the presence of associated factors. Here are some methods of treatment that may be considered for exudative otitis media in a child:

Observation and Monitoring:

In some cases, especially if the fluid accumulation is mild and does not significantly impact the child's hearing or quality of life, a healthcare professional may recommend a period of observation and monitoring. EOM can resolve on its own over time.

Management of Underlying Factors:

Addressing underlying factors contributing to EOM can be important. For example, managing allergies or respiratory infections may help reduce inflammation and fluid buildup in the middle ear.

Nasal Steroids:

Nasal steroids may be recommended to reduce nasal congestion and inflammation, especially if EOM is associated with conditions like allergic rhinitis. These medications can help improve Eustachian tube function and ventilation of the middle ear.

Antibiotics:

While EOM is not typically caused by bacterial infection, if there is evidence of bacterial infection or if EOM is associated with acute otitis media, a healthcare professional may prescribe antibiotics. Antibiotics are not the primary treatment for EOM, but they may be used in specific cases.

Autoinflation (Valsalva Maneuver):

In some cases, healthcare providers may recommend autoinflation exercises, also known as the Valsalva maneuver. This involves encouraging the child to perform actions that help equalize pressure in the middle ear, such as blowing up a balloon.

Tympanostomy Tubes (Ear Tubes):

For recurrent or persistent cases of EOM, where fluid continues to accumulate and impact hearing, healthcare providers may recommend the insertion of tympanostomy tubes. These tiny tubes are placed through the eardrum to allow ventilation and drainage of the middle ear.

Myringotomy:

Myringotomy is a surgical procedure where a small incision is made in the eardrum to drain fluid from the middle ear. This may be performed in conjunction with the placement of tympanostomy tubes.

Treatment decisions are individualized based on the child's specific circumstances, the severity of symptoms, and the duration of the condition. It's important for parents or caregivers to work closely with healthcare professionals to determine the most appropriate treatment plan for their child's exudative otitis media. Regular follow-up visits are typically recommended to monitor the child's progress and adjust the treatment plan as needed.

18. What is the result of a verbal audiometry test and how to interpret it?

Verbal audiometry is a type of hearing test that assesses an individual's ability to understand and repeat spoken words or phrases. The results of a verbal audiometry test are typically presented in the form of a percentage score, indicating the percentage of words or phrases correctly repeated by the individual being tested.

Here's how to interpret the results of a verbal audiometry test:

Word Recognition Score (WRS):

The percentage score obtained in a verbal audiometry test is often referred to as the Word Recognition Score (WRS) or Speech Discrimination Score. It represents the proportion of words or phrases presented to the individual that were correctly identified and repeated.

Percentage Ranges:

The percentage can range from 0% to 100%, with 100% indicating perfect word recognition. The results are typically categorized into different ranges:

Normal Range (90-100%): Excellent word recognition.

Mild Hearing Loss (80-89%): Slight difficulty with word recognition.

Moderate Hearing Loss (70-79%): Moderate difficulty with word recognition.

Severe Hearing Loss (50-69%): Significant difficulty with word recognition.

Profound Hearing Loss (<50%): Poor word recognition.

Configuration of Hearing Loss:

The pattern of word recognition scores can provide information about the configuration of hearing loss. For example, if there is a significant difference in scores across different frequencies, it may suggest a specific pattern of hearing loss.

Use of Phonetically Balanced Word Lists:

Verbal audiometry tests often use phonetically balanced word lists, which include words that contain a representative sample of speech sounds. This helps ensure that the test accurately reflects an individual's ability to understand everyday speech.

Consideration of Context:

It's important to consider the overall context of the individual's hearing ability. For example, someone with a mild hearing loss may still have good word recognition in a quiet environment but may struggle more in noisy or challenging listening situations.

Interpreting the results of a verbal audiometry test should be done by a qualified audiologist or hearing healthcare professional. They will consider the entire audiometric profile, including pure-tone audiometry results and other relevant tests, to provide a comprehensive understanding of the individual's hearing abilities. The results help guide decisions regarding the need for hearing aids, assistive devices, or other interventions to address communication difficulties associated with hearing loss.

19. What are the results of otoscopy for otosclerosis?

Otoscopy alone may not provide definitive results for diagnosing otosclerosis, as it is primarily a clinical examination technique to visualize the external ear canal and the eardrum. Otosclerosis is an inner ear disorder that involves abnormal bone growth in the middle ear, particularly around the stapes bone, which can lead to hearing loss.

To diagnose otosclerosis, a combination of clinical assessments and diagnostic tests is typically used. Here are some aspects of otoscopy and related tests that may be relevant in the context of otosclerosis:

Otoscopy:

During otoscopy, the healthcare provider examines the ear canal and eardrum using an otoscope. Signs that may be observed in otoscopy include a normal or slightly retracted eardrum, and sometimes a bluish hue (Schwartz sign) may be visible due to vascular changes associated with otosclerosis. However, otoscopy alone cannot definitively diagnose otosclerosis.

Audiometry:

Pure-tone audiometry is a key diagnostic test for otosclerosis. Individuals with otosclerosis often exhibit a characteristic pattern of conductive hearing loss, particularly at low frequencies. The air-bone gap on the audiogram may be indicative of otosclerosis, where bone conduction thresholds are within the normal range, but air conduction thresholds are elevated.

Tympanometry:

Tympanometry measures the mobility of the eardrum and middle ear system. In otosclerosis, tympanometry may show a characteristic pattern, such as a normal compliance but a reduced ear canal volume. Tympanometry, along with audiometry, contributes to the diagnosis of otosclerosis.

Stapedial Reflex Testing:

Stapedial reflex testing involves measuring the reflex contraction of the stapedius muscle in response to loud sounds. Individuals with otosclerosis may have absent or elevated stapedial reflex thresholds.

Imaging Studies:

High-resolution imaging techniques like temporal bone CT scans can provide detailed images of the middle ear structures. Imaging may reveal specific features associated with otosclerosis, such as the characteristic "double-ring" appearance around the stapes on a CT scan.

Ultimately, the diagnosis of otosclerosis is often made based on a combination of clinical history, physical examination, audiometric testing, and imaging studies. If otosclerosis is suspected, an otolaryngologist or an ear, nose, and throat (ENT) specialist may be consulted for further evaluation and management. Surgical intervention, such as stapedectomy, is a common treatment option for individuals with symptomatic otosclerosis.

20. What implantable hearing prostheses can be used in children?

Several implantable hearing prostheses can be used in children with hearing loss. The choice of a specific device depends on factors such as the type and degree of hearing loss, the child's age, and individual factors. Here are some of the implantable hearing prostheses commonly used in children:

Cochlear Implants:

Cochlear implants are electronic devices that are surgically implanted to provide direct electrical stimulation to the auditory nerve. They are suitable for children with severe to profound sensorineural hearing loss who do not benefit significantly from traditional hearing aids. Cochlear implants can be used in infants and older children.

Bone-Anchored Hearing Aids (BAHA):

Bone-Anchored Hearing Aids (BAHA) are surgically implanted devices that use bone conduction to transmit sound vibrations directly to the inner ear. They are suitable for children with conductive or mixed hearing loss or single-sided deafness. BAHA devices can be implanted in older children once the skull has matured, and there are also options for non-surgical BAHA solutions.

Auditory Brainstem Implants (ABI):

Auditory Brainstem Implants (ABI) are implanted devices designed for individuals with a missing or damaged cochlear nerve, which may occur in cases of bilateral cochlear nerve deficiency. ABIs are more commonly used in children with neurofibromatosis type II (NF2).

Middle Ear Implants:

Middle ear implants are surgically placed in the middle ear and are designed to enhance sound transmission through the natural hearing pathway. These devices can be considered for certain types of sensorineural or mixed hearing loss in children.

Vibrant Soundbridge (VSB):

The Vibrant Soundbridge is a type of middle ear implant that directly drives the middle ear structures. It is suitable for children with sensorineural or mixed hearing loss and can be implanted in children as young as 5 years old.

Earlens Contact Hearing Solution:

The Earlens Contact Hearing Solution is a non-surgical, light-driven hearing aid system that uses a lens placed on the eardrum to directly vibrate it. This system is suitable for children with mild to moderately severe sensorineural hearing loss.

The selection of an implantable hearing prosthesis is based on a thorough evaluation by an audiologist, otolaryngologist (ENT specialist), and other relevant healthcare professionals. The child's age, hearing profile, medical history, and individual needs are carefully considered to determine the most appropriate device.

It's important for parents and caregivers to work closely with the healthcare team to make informed decisions about the type of implantable hearing prosthesis that will best meet the child's needs and provide optimal auditory outcomes.

ChatGPT's answers (to questions posed in English), translated into Polish using DeepL (note that the questions are the same as in the original Polish version)

1. **Czym jest system CROS?**

System aparatów słuchowych CROS to rozwiązanie zaprojektowane, aby pomóc osobom z jednostronnym ubytkiem słuchu. Jednostronny ubytek słuchu oznacza, że dana osoba ma znaczny ubytek słuchu w jednym uchu, podczas gdy drugie ucho ma normalny lub prawie normalny słuch. System CROS jest szczególnie przydatny dla osób, które nie słyszą na jedno ucho.

"CROS" to skrót od Contralateral Routing of Signals. Oto jak zazwyczaj działa system aparatów słuchowych CROS:

Mikrofon po stronie osoby niesłyszącej: Mikrofon jest umieszczany po stronie głowy z ubytkiem słuchu. Mikrofon ten odbiera dźwięki z głuchego lub niedosłyszącego ucha.

Transmisja do lepszego ucha: Przechwycony dźwięk jest bezprzewodowo przesyłany do aparatu słuchowego lub odbiornika znajdującego się po stronie lepiej słyszącej. Pozwala to osobie słyszeć dźwięki z obu stron.

Zwiększona świadomość: Przesyłając dźwięki ze strony niedosłyszącej do lepiej słyszącego ucha, system CROS pomaga osobom z jednostronnym ubytkiem słuchu być bardziej świadomymi otaczającego ich środowiska i lepiej rozumieć kierunkowość dźwięków.

Technologia ta różni się od tradycyjnych aparatów słuchowych, które są przeznaczone do wzmacniania dźwięków u osób z ubytkiem słuchu w obu uszach. System aparatów słuchowych CROS jest specjalnie dostosowany dla osób z jednostronnym ubytkiem słuchu, zapewniając bardziej kompleksowe wrażenia słuchowe.

2. **Czym jest niedosłuch przewodzeniowy?**

Przewodzeniowy ubytek słuchu jest rodzajem upośledzenia słuchu, które występuje, gdy występuje problem z transmisją fal dźwiękowych z ucha zewnętrznego lub środkowego do ucha wewnętrznego. Ten rodzaj ubytku słuchu może wynikać z różnych przyczyn, które wpływają na zdolność ucha do skutecznego przewodzenia dźwięku.

Najczęstsze przyczyny przewodzeniowego ubytku słuchu obejmują:

Infekcje ucha: Infekcje przewodu słuchowego lub ucha środkowego mogą powodować stan zapalny i gromadzenie się płynu, wpływając na ruch błony bębenkowej i małych kości (kosteczek słuchowych) w uchu środkowym.

Zablokowanie przewodu słuchowego: Nagromadzenie woskowiny (woskowiny) lub obecność ciała obcego w uchu może blokować dotarcie fal dźwiękowych do błony bębenkowej.

Perforacja błony bębenkowej: Rozdarcie lub dziura w błonie bębenkowej może zakłócić normalną transmisję dźwięku.

Otoskleroza: Nieprawidłowy wzrost kości w uchu środkowym może zakłócać ruch kości strzemiączka, jednego z trzech kosteczek słuchowych, prowadząc do przewodzeniowego ubytku słuchu.

Wady rozwojowe ucha: Niektóre osoby mogą urodzić się z nieprawidłowościami strukturalnymi w uchu zewnętrznym lub środkowym, które powodują przewodzeniowy ubytek słuchu.

Przewodzeniowy ubytek słuchu jest na ogół uleczalny, a w wielu przypadkach interwencja medyczna lub zabiegi chirurgiczne mogą pomóc w przywróceniu lub poprawie słuchu. Opcje leczenia mogą obejmować usunięcie woskowiny usznej, leczenie antybiotykami infekcji, chirurgiczną naprawę perforowanej błony bębenkowej lub procedury korygujące nieprawidłowości w uchu środkowym.

Należy zauważyć, że przewodzeniowy ubytek słuchu różni się od odbiorczego ubytku słuchu, który wiąże się z uszkodzeniem ucha wewnętrznego lub nerwu słuchowego i często jest nieodwracalny. W niektórych przypadkach osoby mogą doświadczać kombinacji przewodzeniowego i odbiorczego ubytku słuchu, znanego jako mieszany ubytek słuchu. W przypadku podejrzenia ubytku słuchu zaleca się konsultację z audiologiem lub pracownikiem służby zdrowia w celu przeprowadzenia kompleksowej oceny słuchu.

3. Jakie częstotliwości odpowiadają za odbiór dźwięków mowy u dzieci?

Dźwięki mowy obejmują zakres częstotliwości, a różne częstotliwości przyczyniają się do percepcji różnych dźwięków mowy. Ogólnie rzecz biorąc, częstotliwości związane z dźwiękami mowy u dzieci (a także u dorosłych) mieszczą się w zakresie ludzkiego słuchu, który wynosi około 20 Hz do 20 000 Hz. Jednak częstotliwości kluczowe dla rozumienia mowy znajdują się zazwyczaj w zakresie średnich częstotliwości.

Podstawowa częstotliwość (lub wysokość) ludzkiej mowy, która ma kluczowe znaczenie dla przekazywania intonacji i emocjonalnych aspektów mowy, zwykle mieści się w zakresie od 125 Hz do 250 Hz. Samogłoski w mowie charakteryzują się częstotliwościami formantowymi, przy czym pierwszy formant zwykle wynosi od około 300 Hz do 700 Hz, a drugi formant od około 800 Hz do 1200 Hz.

Z drugiej strony spółgłoski obejmują szerszy zakres częstotliwości. Niektóre składowe mowy o wyższej częstotliwości związane z dźwiękami spółgłoskowymi mogą przekraczać 2000 Hz.

Ważne jest, aby pamiętać, że percepcja mowy obejmuje integrację informacji na różnych częstotliwościach, a konkretne częstotliwości, które przyczyniają się do zrozumienia dźwięków mowy, mogą się różnić w zależności od cech językowych i akustycznych używanego języka.

W kontekście oceny i leczenia ubytku słuchu u dzieci, audiolodzy często koncentrują się na zakresie częstotliwości związanym z dźwiękami mowy, aby zapewnić, że urządzenia wzmacniające (takie jak aparaty słuchowe) zapewniają wystarczające wzmocnienie w tych krytycznych obszarach częstotliwości dla skutecznego rozumienia mowy i rozwoju języka.

4. Jakie mogą być przyczyny zapalenia ucha u dziecka?

Zapalenie ucha, które odnosi się do zapalenia ucha, może objawiać się w różnych formach. Dwa główne typy zapalenia ucha u dzieci to zapalenie ucha środkowego i zapalenie ucha zewnętrznego, z których każdy ma swój własny zestaw przyczyn:

Zapalenie ucha środkowego:

Infekcja bakteryjna lub wirusowa: Zapalenie ucha środkowego jest często związane z infekcjami wywołanymi przez bakterie lub wirusy. Infekcje układu oddechowego, takie jak przeziębienie, mogą prowadzić do gromadzenia się płynu w uchu środkowym, tworząc środowisko sprzyjające rozwojowi bakterii lub wirusów.

Dysfunkcja trąbki Eustachiusza: Trąbki Eustachiusza, które łączą ucho środkowe z tylną częścią gardła, pomagają regulować ciśnienie powietrza w uchu. Dysfunkcja tych trąbek, często spowodowana czynnikami anatomicznymi lub stanem zapalnym, może prowadzić do gromadzenia się płynu i zapalenia ucha środkowego.

Alergie: Reakcje alergiczne mogą przyczyniać się do zapalenia i przekrwienia trąbek Eustachiusza, zwiększając ryzyko zapalenia ucha środkowego.

Czynniki środowiskowe: Narażenie na bierne palenie lub zanieczyszczenia może być czynnikiem ryzyka infekcji ucha u dzieci.

Zapalenie ucha zewnętrznego (ucho pływaka):

Wilgoć i wilgotność: Zapalenie ucha zewnętrznego jest często związane z nadmiarem wilgoci w przewodzie słuchowym, co tworzy środowisko sprzyjające rozwojowi bakterii lub grzybów. Może to być bardziej powszechne w ciepłych i wilgotnych warunkach.

Uraz lub podrażnienie: Drapanie przewodu słuchowego przedmiotami takimi jak waciki lub wkładanie ciał obcych może uszkodzić delikatną skórę przewodu słuchowego, prowadząc do stanu zapalnego.

Nadmiar woskowiny: Nagromadzenie woskowiny usznej może stworzyć korzystne środowisko dla rozwoju bakterii i przyczynić się do zapalenia ucha zewnętrznego.

Środki zapobiegawcze w przypadku obu rodzajów zapalenia ucha u dzieci obejmują przestrzeganie zasad higieny, unikanie narażenia na dym i leczenie alergii, jeśli występuje. Szybkie leczenie infekcji dróg oddechowych i unikanie długotrwałej ekspozycji na wodę w uchu może pomóc w zapobieganiu infekcjom ucha.

Jeśli dziecko wykazuje objawy infekcji ucha, takie jak ból ucha, drenaż lub zmiany słuchu, ważne jest, aby skonsultować się z lekarzem w celu właściwej diagnozy i leczenia. Leczenie może obejmować antybiotyki w przypadku infekcji bakteryjnych, leczenie bólu, a w niektórych przypadkach umieszczenie przewodów słuchowych w przypadku nawracającego zapalenia ucha środkowego.

5. Jak zinterpretować badanie audiometrii tonalnej, czym jest norma słyszenia?

Audiometria tonalna to badanie słuchu, które mierzy zdolność danej osoby do słyszenia dźwięków o różnych częstotliwościach i natężeniach. Wyniki audiometrii tonalnej są zazwyczaj przedstawiane na audiogramie, który jest wykresem pokazującym progi słyszenia danej osoby przy różnych częstotliwościach. Oto jak interpretować wyniki audiometrii tonalnej:

Podstawy audiogramu:

Oś pozioma audiogramu przedstawia częstotliwości, zwykle od niskich do wysokich (mierzone w hercach, Hz).

Oś pionowa reprezentuje intensywność lub głośność, mierzoną w decybelach (dB). Górna część audiogramu reprezentuje głośne dźwięki, a dolna - ciche.

Symbole na audiogramie:

O (kółko): Reprezentuje prawe ucho.

X (krzyżyk): Reprezentuje lewe ucho.

Progi audiometryczne: Punkty na wykresie, w których naniesione są symbole, wskazują najcichsze dźwięki, które dana osoba może usłyszeć dla każdej częstotliwości. Im niższy próg, tym lepszy słuch.

Stopnie utraty słuchu:

Normalny słuch: Progi na poziomie lub poniżej 20 dB dla wszystkich częstotliwości są uważane za mieszczące się w normalnym zakresie.

Łagodny ubytek słuchu: Progi między 20 a 40 dB.

Umiarkowany ubytek słuchu: Progi między 40 a 60 dB.

Poważny ubytek słuchu: Progi między 60 a 80 dB.

Głęboki ubytek słuchu: progi powyżej 80 dB.

Konfiguracja ubytku słuchu:

Kształt lub wzór audiogramu może dostarczyć informacji o konfiguracji ubytku słuchu (np. płaski, opadający, wznoszący się).

Częstotliwości mowy:

Ważne częstotliwości mowy są zazwyczaj badane przy 500 Hz, 1000 Hz, 2000 Hz, 4000 Hz i 8000 Hz.

Należy pamiętać, że interpretacja wyników audiometrii tonalnej powinna być przeprowadzona przez wykwalifikowanego audiologa lub protetyka słuchu. Weźmie on pod uwagę cały audiogram, oceni oboje uszu i przedstawi odpowiednie zalecenia w oparciu o profil słuchu danej osoby.

Jeśli masz konkretny wynik audiogramu i chciałbyś uzyskać bardziej szczegółowe informacje, zalecamy konsultację z audiologiem, ponieważ może on udzielić spersonalizowanych wskazówek w oparciu o konkretny kontekst i potrzeby danej osoby.

6. Jak wygląda badanie ABR u dziecka?

Test słuchowej odpowiedzi pnia mózgu (ABR), znany również jako słuchowa odpowiedź wywołana pnia mózgu (BAER), jest testem diagnostycznym stosowanym do oceny nerwu słuchowego i odpowiedzi pnia mózgu na dźwięk. Jest on powszechnie stosowany zarówno u dorosłych, jak i dzieci, w tym niemowląt i noworodków. U dzieci test ABR jest szczególnie przydatny, gdy tradycyjne behawioralne testy słuchu są trudne do przeprowadzenia ze względu na wiek lub czynniki rozwojowe.

Oto jak działa test ABR:

Umieszczenie elektrod: Małe elektrody są umieszczane na skórze głowy dziecka, a czasami na płatkach uszu. Elektrody te odbierają odpowiedzi elektryczne generowane przez nerw słuchowy i pień mózgu w odpowiedzi na bodźce dźwiękowe.

Prezentacja bodźców: Dziecko jest narażone na serię krótkich dźwięków klikania lub impulsów tonowych przez słuchawki. Dźwięki te są prezentowane z różną intensywnością i częstotliwością.

Pomiar odpowiedzi: Elektrody odbierają odpowiedzi elektryczne generowane przez nerw słuchowy i pień mózgu w wyniku prezentowanych dźwięków. Odpowiedzi są następnie rejestrowane i analizowane.

Określenie progu: Badanie ABR może dostarczyć informacji na temat progów słyszenia dziecka, czyli najcichszych dźwięków, jakie jest ono w stanie wykryć.

Test ABR jest cenny w kilku scenariuszach, w tym:

Badanie przesiewowe słuchu noworodków: Jest on często wykorzystywany jako część programów badań przesiewowych słuchu noworodków w celu identyfikacji problemów ze słuchem we wczesnym okresie niemowlęcym.

Małe dzieci: Gdy tradycyjne testy behawioralne stanowią wyzwanie ze względu na wiek lub etap rozwoju dziecka.

Ocena neurologiczna: Może dostarczyć informacji na temat integralności nerwu słuchowego i ścieżek pnia mózgu.

Test ABR jest nieinwazyjnym i obiektywnym pomiarem funkcji słuchu. Chociaż nie dostarcza informacji na temat percepcji mowy lub rozwoju językowego dziecka, jest niezbędnym narzędziem w kompleksowej ocenie układu słuchowego dziecka. Wyniki testu ABR mogą pomóc w dalszej diagnostyce i interwencji, jeśli zidentyfikowane zostaną problemy ze słuchem. Wykwalifikowany audiolog zazwyczaj przeprowadza i interpretuje test ABR.

7. Na czym polegają przesiewowe badania słuchu u noworodków?

Badania przesiewowe słuchu noworodków to zestaw testów mających na celu identyfikację problemów ze słuchem u niemowląt wkrótce po urodzeniu. Celem badań przesiewowych słuchu noworodków jest wczesne wykrycie ubytku słuchu, tak aby można było szybko rozpocząć interwencję i leczenie, promując optymalny rozwój języka i mowy. Wczesne wykrycie ma kluczowe znaczenie, ponieważ niemowlęta uczą się języka i umiejętności komunikacyjnych w pierwszych miesiącach i latach życia, a słuch ma fundamentalne znaczenie dla tego procesu.

Istnieją dwa główne rodzaje badań przesiewowych słuchu u noworodków:

Test emisji otoakustycznych (OAE): W tym teście mała sonda jest umieszczana w uchu dziecka i emituje dźwięk. Jeśli ucho wewnętrzne dziecka (ślimak) funkcjonuje prawidłowo, będzie ono wytwarzać odpowiedź podobną do echa, zwaną emisją otoakustyczną. Obecność tych emisji sugeruje prawidłowy słuch.

Test słuchowej odpowiedzi pnia mózgu (ABR): Test ten mierzy aktywność elektryczną nerwu słuchowego i pnia mózgu w odpowiedzi na dźwięk. Małe elektrody są umieszczane na skórze głowy dziecka, a dziecko słucha klikających dźwięków. Test rejestruje odpowiedzi elektryczne, a audiolog analizuje je w celu określenia stanu słuchu dziecka.

Badania przesiewowe słuchu noworodków są zwykle przeprowadzane w szpitalu wkrótce po urodzeniu, często przed wypisaniem dziecka do domu. Testy są szybkie, nieinwazyjne i zazwyczaj nie powodują dyskomfortu u niemowlęcia. Jeśli wstępne badanie przesiewowe sugeruje potencjalny problem ze słuchem, mogą być zalecane dalsze oceny diagnostyczne, takie jak kompleksowa ocena audiologiczna.

Ważne jest, aby pamiętać, że nie wszystkie dzieci, które nie przejdą wstępnego badania przesiewowego, mają trwały ubytek słuchu. Niektóre niemowlęta mogą mieć tymczasowe problemy ze słuchem z powodu czynników takich jak płyn w uchu lub zanieczyszczenia, a dalsza ocena pomaga odróżnić tymczasową od trwałej utraty słuchu.

Wczesna interwencja i odpowiednie wsparcie dla niemowląt z ubytkiem słuchu może znacząco wpłynąć na ich rozwój językowy i komunikacyjny. Jeśli masz obawy dotyczące słuchu swojego dziecka, ważne jest, aby omówić je z pracownikami służby zdrowia, a jeśli to konieczne, wykonać zalecane badania przesiewowe i oceny słuchu.

8. W jaki sposób przebiega wysiękowe zapalenie ucha środkowego u dziecka?

Wysiękowe zapalenie ucha środkowego (EOM), znane również jako surowicze lub wydzielnicze zapalenie ucha środkowego, jest stanem charakteryzującym się gromadzeniem się płynu w uchu środkowym bez objawów ostrej infekcji. Stan ten jest często spotykany u dzieci i może mieć różny przebieg, w zależności od czynników takich jak przyczyna, leczenie i indywidualna reakcja. Oto ogólny przegląd przebiegu wysiękowego zapalenia ucha środkowego u dziecka:

Początek i rozwój: EOM często rozwija się w wyniku dysfunkcji trąbki Eustachiusza, która łączy ucho środkowe z tylną ścianą gardła. Dysfunkcja ta może prowadzić do słabej wentylacji i drenażu ucha środkowego, powodując gromadzenie się płynu.

Bezobjawowe lub łagodne objawy: W niektórych przypadkach dzieci z EOM mogą nie wykazywać zauważalnych objawów lub mogą doświadczać jedynie łagodnych objawów, takich jak uczucie pełności w uchu, łagodny ubytek słuchu lub sporadyczne trzaski.

Utrzymywanie się płynu: Płyn w uchu środkowym może utrzymywać się przez dłuższy czas, prowadząc do przewlekłego lub nawracającego stanu. Na utrzymywanie się płynu mogą mieć wpływ takie czynniki jak alergie, narażenie na bierne palenie lub problemy anatomiczne związane z trąbką Eustachiusza.

Utrata słuchu: Jednym z głównych problemów związanych z EOM jest jego wpływ na słuch. Nagromadzenie płynu w uchu środkowym może zakłócać transmisję dźwięku, powodując przewodzeniowy ubytek słuchu.

Spontaniczne ustąpienie: W niektórych przypadkach EOM może ustąpić samoistnie bez interwencji. W miarę jak dziecko rośnie, a trąbka Eustachiusza dojrzewa, zdolność do utrzymania prawidłowej wentylacji i drenażu ucha środkowego może ulec poprawie, prowadząc do ustąpienia schorzenia.

Opcje leczenia: Gdy EOM utrzymuje się lub powoduje znaczną utratę słuchu, pracownicy służby zdrowia mogą rozważyć interwencje. Opcje leczenia mogą obejmować zarządzanie czynnikami podstawowymi (takimi jak alergie), umieszczenie rurek usznych (rurek tympanostomijnych) w celu umożliwienia drenażu płynu lub inne interwencje medyczne.

Obserwacja i monitorowanie: Dzieci z EOM zazwyczaj przechodzą regularne badania kontrolne w celu monitorowania stanu i oceny słuchu. Pracownicy służby zdrowia mogą dostosować plan leczenia w oparciu o postępy dziecka.

Ważne jest, aby rodzice i opiekunowie ściśle współpracowali z pracownikami służby zdrowia w celu skutecznego leczenia wysiękowego zapalenia ucha środkowego. Regularne monitorowanie i odpowiednie interwencje mogą pomóc zminimalizować wpływ EOM na słuch i ogólne samopoczucie dziecka.

9. **W jaki sposób można zbadać słuch u rocznego dziecka?**

Badanie słuchu u rocznego dziecka wymaga zastosowania specjalistycznych technik dostosowanych do jego wieku i etapu rozwoju. Oto dwie powszechnie stosowane metody oceny słuchu u niemowląt i małych dzieci, w tym rocznych dzieci:

Audiometria obserwacyjna behawioralna (BOA):

Metoda ta polega na obserwacji reakcji behawioralnych dziecka na dźwięk. Podczas gdy dziecko jest zaangażowane w zajęcia lub zabawę, audiolog lub pracownik służby zdrowia prezentuje dźwięki o różnych częstotliwościach i głośności oraz monitoruje reakcje dziecka.

Reakcje mogą obejmować odwracanie przez dziecko głowy w kierunku dźwięku, wykazywanie odruchu przestrachu lub zwiększonej uwagi. Metoda ta jest odpowiednia dla dzieci w wieku zaledwie kilku miesięcy.

Audiometria ze wzmocnieniem wzroku (VRA):

VRA to bardziej ustrukturyzowana metoda, która polega na łączeniu bodźców dźwiękowych z nagrodą wizualną. Dziecko jest warunkowane do kojarzenia dźwięku z nagradzającym bodźcem wizualnym, takim jak podświetlana zabawka lub animowany wyświetlacz. Gdy dziecko reaguje na dźwięk, jest nagradzane wizualnie.

Metoda ta jest często stosowana u dzieci w wieku od sześciu miesięcy do dwóch lat. Dziecko zazwyczaj siedzi na kolanach opiekuna, a głośniki są używane do prezentowania dźwięków z różnych kierunków.

Ważne jest, aby pamiętać, że testowanie słuchu jednorocznego dziecka może wymagać dostosowania i elastyczności w oparciu o zachowanie dziecka i jego zakres uwagi. Środowisko testowe jest często zaprojektowane tak, aby było przyjazne dla dziecka i angażujące, aby zachęcić je do udziału.

Jeśli podczas tych wstępnych badań przesiewowych zostaną zidentyfikowane obawy dotyczące słuchu, mogą być zalecane bardziej kompleksowe testy, takie jak badanie słuchowej odpowiedzi pnia mózgu (ABR). ABR to test neurofizjologiczny, który mierzy aktywność elektryczną nerwu słuchowego i pnia mózgu w odpowiedzi na dźwięk.

Rodzice i opiekunowie odgrywają kluczową rolę w dostarczaniu informacji na temat reakcji dziecka na dźwięk i powinni przekazywać wszelkie wątpliwości pracownikom służby zdrowia. Jeśli podejrzewa się problemy ze słuchem lub jeśli dziecko jest zagrożone utratą słuchu, może być zalecana wczesna interwencja i dalsze badania diagnostyczne w celu rozwiązania wszelkich potencjalnych wyzwań związanych ze słuchem.

10. **Na czym polega badanie audiometrii słownej?**

Audiometria słowna to rodzaj testu słuchu, który ocenia zdolność danej osoby do rozumienia i powtarzania wypowiadanych słów lub fraz. Test ten jest powszechnie stosowany do oceny funkcjonalnych aspektów słuchu, szczególnie w zakresie percepcji mowy i dyskryminacji. Audiometria werbalna jest często wykonywana u osób, które są wystarczająco duże, aby reagować werbalnie, zwykle od około 5 lub 6 roku życia.

Oto przegląd tego, jak zwykle przeprowadzana jest audiometria słowna:

Test rozpoznawania słów: Podczas testu rozpoznawania słów osoba słucha listy słów jednosylabowych lub dwusylabowych prezentowanych na komfortowym poziomie słyszenia. Słowa są zazwyczaj wypowiadane przez audiologa przez słuchawki.

Ocenianie odpowiedzi: Osoba jest następnie proszona o powtórzenie każdego usłyszanego słowa. Audiolog zapisuje odpowiedzi danej osoby, odnotowując, które słowa zostały powtórzone poprawnie, a które zostały źle zrozumiane lub niedokładnie usłyszane.

Procent poprawnych odpowiedzi: Wyniki są wyrażone jako procent poprawnych odpowiedzi. Odsetek ten odzwierciedla zdolność danej osoby do dokładnego rozpoznawania i powtarzania prezentowanych słów.

Dyskryminacja mowy: Oprócz testu rozpoznawania słów można przeprowadzić test dyskryminacji mowy. Obejmują one prezentację zdań lub fraz, a dana osoba proszona jest o ich powtórzenie. Ten rodzaj badania dostarcza informacji na temat zdolności danej osoby do rozumienia mowy w bardziej złożonym kontekście.

Audiometria słowna jest ważnym elementem kompleksowej oceny audiologicznej, ponieważ ocenia, jak dobrze dana osoba może rozumieć i komunikować się za pomocą języka mówionego. Wyniki audiometrii słownej, wraz z innymi testami, takimi jak audiometria czysto-tonowa i tympanometria, przyczyniają się do ogólnego zrozumienia zdolności słuchowych danej osoby i mogą kierować zaleceniami dotyczącymi interwencji, takimi jak aparaty słuchowe lub inne urządzenia wspomagające.

Ważne jest, aby pamiętać, że audiometria słowna jest zwykle stosowana u osób, które mają wystarczające umiejętności językowe i poznawcze, aby wziąć udział w teście. W przypadku młodszych dzieci lub osób z wyzwaniami rozwojowymi lub komunikacyjnymi do oceny zdolności słyszenia można zastosować alternatywne metody, takie jak audiometria zabawowa lub audiometria ze wzmocnieniem wizualnym.

11. **Jakie typy tympanogramów wyróżniamy w audiometrii impedancyjnej?**

Tympanometria to badanie wykorzystywane w audiometrii impedancyjnej do oceny ruchomości błony bębenkowej i układu ucha środkowego. Test mierzy podatność ucha środkowego na zmiany ciśnienia powietrza w przewodzie słuchowym. Tympanogramy są graficzną reprezentacją wyników i pomagają audiologom zdiagnozować różne schorzenia ucha środkowego. Istnieje kilka rodzajów tympanogramów i są one podzielone na kategorie w oparciu o ich kształt i charakterystykę:

Tympanogram typu A: Jest to normalny tympanogram. Pokazuje szczyt zgodności w połowie badanego zakresu ciśnienia. Wartość szczytowa wskazuje, że błona bębenkowa i układ ucha środkowego funkcjonują prawidłowo i występuje normalny ruch w odpowiedzi na zmiany ciśnienia powietrza.

Tympanogram typu As: Tympanogram typu As ma zmniejszoną podatność szczytową, co sugeruje zmniejszoną ruchomość błony bębenkowej. Ten wzorzec jest często związany z usztywnieniem układu ucha środkowego, które może wystąpić w stanach takich jak otoskleroza lub częściowe przerwanie ciągłości kosteczek słuchowych.

Tympanogram typu Ad: Tympanogram typu Ad również charakteryzuje się zmniejszoną podatnością szczytową, ale z nieprawidłowo głęboką objętością przewodu słuchowego. Ten wzorzec jest często związany z wiotką lub hipermobilną błoną bębenkową, co można zaobserwować w stanach takich jak niedrożność trąbki Eustachiusza.

Tympanogram typu B: Tympanogram typu B nie wykazuje wyraźnego szczytu i jest płaski w całym zakresie ciśnienia. Ten wzór może wskazywać na brak ruchomości błony bębenkowej, często obserwowany w stanach takich jak wysięk w uchu środkowym (płyn w uchu środkowym) lub perforacja błony bębenkowej.

Tympanogram typu C: Tympanogram typu C ma szczytową zgodność przesuniętą w stronę ujemnego ciśnienia na wykresie, co wskazuje na ujemne ciśnienie w uchu środkowym. Ten wzór jest często związany z dysfunkcją trąbki Eustachiusza lub zatkaniem, gdzie ciśnienie w uchu środkowym jest niższe niż normalnie.

Te typy tympanogramów dostarczają cennych informacji na temat stanu ucha środkowego, funkcji trąbki Eustachiusza i potencjalnych problemów wpływających na słuch. Tympanometria jest często wykonywana jako część kompleksowej oceny audiologicznej w celu oceny ogólnego stanu ucha środkowego i przyczynia się do diagnozowania i leczenia różnych schorzeń ucha.

12. W jakim celu wykonuje się wycisk ucha u dzieci?

Wyciski uszu, znane również jako wkładki uszne lub wyciski uszu, obejmują wykonanie formy lub wycisku zewnętrznego kształtu ucha i kanału słuchowego. Wyciski te są wykonywane na zamówienie dla danej osoby i są wykorzystywane do różnych celów. W kontekście dzieci, wyciski uszu mogą być wykonywane w następujących celach:

Dopasowanie aparatów słuchowych: Wyciski uszu są powszechnie stosowane podczas dopasowywania niestandardowych aparatów słuchowych dla dzieci. Niestandardowe aparaty słuchowe są zaprojektowane tak, aby pasowały do unikalnego kształtu i rozmiaru ucha dziecka, zapewniając wygodne i bezpieczne dopasowanie. Wyciski pozwalają protetykowi słuchu na stworzenie aparatów słuchowych dostosowanych do anatomii ucha dziecka.

Wkładka dla ochrony słuchu: Dzieci mogą wymagać ochrony słuchu w hałaśliwym otoczeniu, takim jak koncerty, imprezy sportowe lub głośne zajęcia rekreacyjne. Niestandardowe wkładki uszne mogą być tworzone na podstawie odcisków uszu, aby zapewnić dokładne i skuteczne dopasowanie, oferując spersonalizowaną ochronę słuchu.

Monitory douszne dla muzyków: Jeśli dziecko zajmuje się muzyką, w szczególności występami na żywo, na podstawie odcisków uszu można stworzyć niestandardowe monitory douszne. Monitory te zapewniają wygodne i bezpieczne dopasowanie, dostarczając wysokiej jakości dźwięk, jednocześnie chroniąc uszy przed nadmiernym hałasem.

Zatyczki do pływania lub ochrona przed wodą: Niektóre dzieci mogą skorzystać z niestandardowych zatyczek do pływania lub wkładek chroniących przed wodą. Są one zaprojektowane tak, aby chronić uszy przed wodą podczas takich czynności jak pływanie lub kąpiel. Odciski uszu pomagają stworzyć precyzyjne dopasowanie, aby zapobiec przedostawaniu się wody do kanału słuchowego.

Słuchawki komunikacyjne: W niektórych sytuacjach, takich jak w przypadku dzieci z ubytkiem słuchu, które korzystają z urządzeń komunikacyjnych, mogą być potrzebne niestandardowe formowane wkładki douszne. Te wkładki douszne mogą być tworzone na podstawie odcisków uszu, aby zapewnić wygodne i skuteczne dopasowanie do urządzeń komunikacyjnych.

Należy pamiętać, że wykonywanie wycisków uszu u dzieci wymaga delikatnego i przyjaznego podejścia. Specjaliści, tacy jak audiolodzy lub protetycy słuchu, mający doświadczenie w pracy z dziećmi, mogą zapewnić, że proces ten jest wygodny i bezstresowy dla dziecka.

Ogólnie rzecz biorąc, wyciski uszne dla dzieci służą różnym celom, wszystkie mają na celu poprawę ich doświadczeń słuchowych, ochronę słuchu i poprawę ogólnej jakości życia w różnych środowiskach.

13. Jak w audiogramie oznaczyć przewodnictwo powietrzne?

Badanie przewodnictwa powietrznego na audiogramie służy do oceny zdolności danej osoby do słyszenia dźwięków przenoszonych przez powietrze. Audiogram to wykres przedstawiający najcichsze dźwięki, jakie dana osoba może usłyszeć przy różnych częstotliwościach (wysokości) i natężeniach (głośności). Badanie przewodnictwa powietrznego wymaga użycia słuchawek lub wkładek, a dana osoba reaguje na różne dźwięki prezentowane przez audiologa. Oto jak zazwyczaj przeprowadzane jest badanie przewodnictwa powietrznego:

Sprzęt audiometryczny: Audiolog używa specjalistycznego sprzętu audiometrycznego, w tym skalibrowanych słuchawek lub wkładek i audiometru. Audiometr generuje czyste tony o różnych częstotliwościach i natężeniach.

Wybór częstotliwości: Audiolog wybiera określone częstotliwości do przetestowania. Standardowe częstotliwości audiometryczne obejmują 250 Hz, 500 Hz, 1000 Hz, 2000 Hz, 4000 Hz i 8000 Hz. Częstotliwości te są mierzone w hercach (Hz).

Poziomy intensywności: Dla każdej wybranej częstotliwości audiolog prezentuje dźwięki o różnych poziomach natężenia, mierzonych w decybelach poziomu słyszenia (dB HL). Intensywność zaczyna się od poziomu, który jest zazwyczaj łatwy do usłyszenia, a następnie zmniejsza się w krokach co 5 lub 10 dB, aż do określenia najcichszego dźwięku, jaki dana osoba może usłyszeć.

Reakcja pacjenta: Osoba badana reaguje na każdy dźwięk, naciskając przycisk, podnosząc rękę lub wysyłając inny z góry określony sygnał, gdy usłyszy dźwięk. Odpowiedzi są rejestrowane przez audiologa.

Wykreślanie audiogramu: Wyniki są wykreślane na audiogramie, z częstotliwością na osi poziomej i natężeniem (dB HL) na osi pionowej. Symbole na audiogramie reprezentują najcichsze dźwięki, jakie dana osoba może usłyszeć przy każdej badanej częstotliwości.

Określenie progu: Najcichszy poziom natężenia dźwięku, przy którym dana osoba słyszy każdą częstotliwość, nazywany jest progiem słyszenia. Progi słyszenia są wykorzystywane do oceny stopnia ubytku słuchu dla różnych częstotliwości.

Badanie przewodnictwa powietrznego ocenia cały układ słuchowy, od ucha zewnętrznego do ucha wewnętrznego i dalej. Dostarcza informacji na temat ogólnej wrażliwości słuchu danej osoby i pomaga zidentyfikować rodzaj i konfigurację ubytku słuchu. Jeśli ubytek słuchu zostanie zidentyfikowany, można przeprowadzić dalsze testy, takie jak badanie przewodnictwa kostnego, w celu określenia konkretnego miejsca uszkodzenia słuchu w układzie słuchowym.

14. Co należy zrobić w przypadku nieprawidłowego wyniku przesiewowych badań słuchu u noworodka?

Jeśli wyniki badania przesiewowego słuchu noworodka są nieprawidłowe, ważne jest, aby niezwłocznie wykonać dodatkowe badania i ocenę. Wczesne wykrycie ubytku słuchu ma kluczowe znaczenie dla interwencji i wsparcia w celu zapewnienia optymalnego rozwoju języka i komunikacji. Oto ogólne kroki, które należy podjąć, gdy wynik badania przesiewowego słuchu noworodka jest nieprawidłowy:

Powtórzenie badania przesiewowego: W niektórych przypadkach może wystąpić fałszywie dodatni wynik z powodu takich czynników, jak zanieczyszczenia w przewodzie słuchowym lub niepokój niemowlęcia podczas pierwszego badania przesiewowego. Przed przystąpieniem do dodatkowych testów często powtarza się badanie przesiewowe słuchu w celu potwierdzenia wyniku.

Diagnostyczne badanie słuchowej odpowiedzi pnia mózgu (ABR): Jeśli powtórne badanie przesiewowe nadal wskazuje na potencjalny problem ze słuchem, kolejnym krokiem jest często diagnostyczne badanie słuchowej odpowiedzi pnia mózgu (ABR). ABR to bardziej kompleksowy i szczegółowy test, który mierzy aktywność elektryczną nerwu słuchowego i pnia mózgu w odpowiedzi na dźwięk. Dostarcza informacji na temat obecności, stopnia i rodzaju ubytku słuchu.

Ocena medyczna: Jeśli test ABR potwierdzi ubytek słuchu, niemowlę powinno zostać poddane ocenie medycznej przez otolaryngologa (specjalistę od uszu, nosa i gardła) lub audiologa dziecięcego. Ocena medyczna pomaga zidentyfikować wszelkie podstawowe schorzenia lub kwestie anatomiczne, które mogą przyczyniać się do utraty słuchu.

Planowanie interwencji: Po określeniu rodzaju i stopnia ubytku słuchu można opracować plan interwencji. Plan ten może obejmować zalecenia dotyczące aparatów słuchowych, implantów ślimakowych (w stosownych przypadkach) i usług wczesnej interwencji w celu wsparcia komunikacji i rozwoju językowego dziecka.

Wsparcie i edukacja rodziny: Wspieranie rodziny jest istotnym aspektem radzenia sobie z ubytkiem słuchu u noworodka. Rodziny powinny otrzymywać informacje i zasoby, które pomogą im zrozumieć konsekwencje ubytku słuchu oraz sposoby wspierania komunikacji i rozwoju dziecka. Często dostępne są programy edukacyjne i zasoby dla rodzin z dziećmi niesłyszącymi lub niedosłyszącymi.

Monitorowanie: Regularne wizyty kontrolne i monitorowanie mają kluczowe znaczenie dla oceny postępów dziecka i wprowadzania wszelkich niezbędnych zmian w planie interwencji. Bieżące oceny audiologiczne pomagają śledzić zmiany w słuchu i zapewniają, że odpowiednie interwencje są wdrażane wraz z rozwojem dziecka.

Ważne jest, aby rodzice i opiekunowie aktywnie uczestniczyli w tym procesie, otwarcie komunikowali się z pracownikami służby zdrowia i szukali wsparcia w odpowiednich usługach i organizacjach specjalizujących się we wczesnej interwencji w przypadku ubytku słuchu. Wczesna identyfikacja i interwencja znacząco poprawiają wyniki leczenia dzieci z ubytkiem słuchu.

15. Co oznaczają żółte i niebieskie certyfikaty w książeczce zdrowia dziecka w kontekście przesiewowych badań słuchu dzieci w Polsce?

Od czasu mojej ostatniej aktualizacji wiedzy w styczniu 2022 r. nie mam konkretnych informacji na temat systemu kodowania kolorami stosowanego w książeczkach zdrowia dzieci w Polsce w odniesieniu do zaświadczeń o badaniach przesiewowych słuchu. Systemy opieki zdrowotnej i procedury administracyjne mogą ewoluować i możliwe, że od tego czasu nastąpiły zmiany lub aktualizacje.

W wielu krajach, w tym w Polsce, książeczki zdrowia lub karty zdrowia dziecka często zawierają informacje związane ze zdrowiem dziecka, szczepieniami, wzrostem i kamieniami milowymi rozwoju. Badania przesiewowe słuchu są istotnym elementem monitorowania zdrowia we wczesnym dzieciństwie, a zaświadczenia mogą być wydawane w celu udokumentowania wyników badań przesiewowych słuchu.

Aby uzyskać dokładne i aktualne informacje na temat konkretnego systemu kodowania kolorami stosowanego w certyfikatach badań przesiewowych słuchu w Polsce, zalecam skontaktowanie się z lokalnymi organami ds. zdrowia, pediatrami lub odpowiednimi rządowymi agencjami ds. zdrowia. Mogą oni udzielić szczegółowych informacji na temat aktualnych praktyk, protokołów i znaczenia różnych kolorów lub kodów na certyfikatach badań przesiewowych słuchu w kontekście dokumentacji zdrowotnej dzieci w Polsce.

16. Jakie są przeciwwskazania do używania aparatów słuchowych na przewodnictwo powietrzne?

Aparaty słuchowe z przewodnictwem powietrznym są powszechnie stosowane do wzmacniania dźwięków i poprawy słyszenia u osób z ubytkiem słuchu. Chociaż są one ogólnie bezpieczne i skuteczne, istnieją określone sytuacje lub warunki, w których stosowanie aparatów słuchowych z przewodnictwem powietrznym może być niewskazane. Przeciwwskazania do stosowania aparatów słuchowych z przewodnictwem powietrznym obejmują:

Obecność ostrych infekcji ucha: Osobom z aktywnymi lub ostrymi infekcjami ucha, takimi jak zapalenie ucha środkowego z drenażem, można zalecić unikanie używania aparatów słuchowych do czasu wyleczenia i ustąpienia infekcji. Wprowadzenie aparatów słuchowych w obecności aktywnej infekcji może potencjalnie pogorszyć stan.

Ciężkie zapalenie ucha zewnętrznego lub zapalenie skóry: Ciężkie zapalenie ucha zewnętrznego (zapalenie przewodu słuchowego) lub zapalenie skóry ucha może być przeciwwskazaniem do stosowania tradycyjnych aparatów słuchowych z przewodnictwem powietrznym. Obecność stanu zapalnego lub otwartych ran może sprawić, że noszenie aparatów słuchowych będzie niewygodne lub zaostrzy istniejący stan.

Drenaż lub zapalenie ucha zewnętrznego ze stanem zapalnym: Stany obejmujące uporczywy drenaż lub zapalenie przewodu słuchowego, takie jak przewlekłe zapalenie ucha zewnętrznego, mogą stanowić przeciwwskazanie do stosowania aparatów słuchowych z przewodnictwem powietrznym. Stany te mogą zakłócać prawidłowe funkcjonowanie i dopasowanie aparatów słuchowych.

Anatomiczne nieprawidłowości lub deformacje: Niektóre osoby mogą mieć anatomiczne nieprawidłowości lub deformacje ucha, które utrudniają prawidłowe dopasowanie konwencjonalnych aparatów słuchowych. W takich przypadkach można zbadać alternatywne rozwiązania słuchowe.

Reakcje alergiczne: Przeciwwskazaniem mogą być reakcje alergiczne na materiały stosowane w aparatach słuchowych (np. obudowę, wkładki uszne lub kleje). W takich przypadkach osoby mogą potrzebować materiałów hipoalergicznych lub innych opcji aparatów słuchowych.

Niezdolność do tolerowania używania aparatów słuchowych: Niektóre osoby mogą odczuwać dyskomfort, podrażnienie lub poczucie przytłoczenia podczas korzystania z aparatów słuchowych. W takich przypadkach ważne jest, aby zająć się konkretnymi kwestiami powodującymi dyskomfort i zbadać alternatywne rozwiązania lub dostosowania.

Bardzo ważne jest, aby osoby z ubytkiem słuchu przeszły kompleksową ocenę słuchu przez audiologa lub protetyka słuchu. Audiolog może ocenić konkretne potrzeby danej osoby, określić rodzaj i stopień ubytku słuchu oraz zalecić odpowiednie rozwiązania słuchowe w oparciu o ogólny stan zdrowia danej osoby i względy anatomiczne. Ostatecznie decyzja o zastosowaniu aparatów słuchowych z przewodnictwem powietrznym lub zbadaniu alternatywnych rozwiązań zależy od indywidualnych okoliczności i wskazówek wykwalifikowanych pracowników służby zdrowia.

17. Jakie są najskuteczniejsze metody leczenia wysiękowego zapalenia ucha środkowego u dziecka?

Wysiękowe zapalenie ucha środkowego (EOM), znane również jako surowicze lub wydzielnicze zapalenie ucha środkowego, jest stanem charakteryzującym się gromadzeniem się płynu w uchu środkowym bez objawów ostrej infekcji. Leczenie EOM u dzieci często zależy od przyczyny i obecności powiązanych czynników. Oto kilka metod leczenia, które można rozważyć w przypadku wysiękowego zapalenia ucha środkowego u dziecka:

Obserwacja i monitorowanie: W niektórych przypadkach, zwłaszcza jeśli nagromadzenie płynu jest łagodne i nie wpływa znacząco na słuch dziecka lub jakość jego życia, pracownik służby zdrowia może zalecić okres obserwacji i monitorowania. EOM może z czasem samoistnie ustąpić.

Zarządzanie czynnikami leżącymi u podstaw: Ważne może być zajęcie się podstawowymi czynnikami przyczyniającymi się do EOM. Na przykład leczenie alergii lub infekcji dróg oddechowych może pomóc zmniejszyć stan zapalny i gromadzenie się płynu w uchu środkowym.

Sterydy donosowe: Sterydy donosowe mogą być zalecane w celu zmniejszenia przekrwienia błony śluzowej nosa i stanu zapalnego, zwłaszcza jeśli EOM jest związany ze stanami takimi jak alergiczny nieżyt nosa. Leki te mogą pomóc poprawić funkcjonowanie trąbki Eustachiusza i wentylację ucha środkowego.

Antybiotyki: Chociaż EOM zwykle nie jest spowodowany infekcją bakteryjną, jeśli istnieją dowody na infekcję bakteryjną lub jeśli EOM jest związany z ostrym zapaleniem ucha środkowego, pracownik służby zdrowia może przepisać antybiotyki. Antybiotyki nie są podstawową metodą leczenia EOM, ale mogą być stosowane w określonych przypadkach.

Autoinflacja (manewr Valsalvy): W niektórych przypadkach pracownicy służby zdrowia mogą zalecić ćwiczenia autoinflacji, znane również jako manewr Valsalvy. Polega to na zachęcaniu dziecka do wykonywania czynności, które pomagają wyrównać ciśnienie w uchu środkowym, takich jak nadmuchiwanie balonu.

Rurki tympanostomijne (rurki uszne): W przypadku nawracających lub uporczywych przypadków EOM, w których płyn nadal się gromadzi i wpływa na słuch, pracownicy służby zdrowia mogą zalecić założenie rurek tympanostomijnych. Te małe rurki są umieszczane przez błonę bębenkową, aby umożliwić wentylację i drenaż ucha środkowego.

Myringotomia: Myringotomia to zabieg chirurgiczny polegający na wykonaniu niewielkiego nacięcia w błonie bębenkowej w celu odprowadzenia płynu z ucha środkowego. Zabieg ten może być wykonywany w połączeniu z umieszczeniem rurek tympanostomijnych.

Decyzje dotyczące leczenia są zindywidualizowane w oparciu o konkretne okoliczności dziecka, nasilenie objawów i czas trwania choroby. Ważne jest, aby rodzice lub opiekunowie ściśle współpracowali z pracownikami służby zdrowia w celu ustalenia najbardziej odpowiedniego planu leczenia wysiękowego zapalenia ucha środkowego u dziecka. Zazwyczaj zaleca się regularne wizyty kontrolne w celu monitorowania postępów dziecka i dostosowania planu leczenia w razie potrzeby..

18. Jak wygląda wynik badania audiometrii słownej i jak go interpretować?

Audiometria słowna to rodzaj testu słuchu, który ocenia zdolność danej osoby do rozumienia i powtarzania wypowiadanych słów lub fraz. Wyniki testu audiometrii słownej są zazwyczaj przedstawiane w formie wyniku procentowego, wskazującego odsetek słów lub fraz poprawnie powtórzonych przez badaną osobę.

Oto jak interpretować wyniki testu audiometrii słownej:

Wynik rozpoznawania słów (WRS): Wynik procentowy uzyskany w teście audiometrii słownej jest często określany jako Word Recognition Score (WRS) lub Speech Discrimination Score. Reprezentuje on odsetek słów lub fraz przedstawionych danej osobie, które zostały prawidłowo zidentyfikowane i powtórzone.

Zakresy procentowe: Wartość procentowa może wynosić od 0% do 100%, przy czym 100% oznacza doskonałe rozpoznawanie słów. Wyniki są zazwyczaj podzielone na różne zakresy:

- Zakres normalny (90-100%): Doskonałe rozpoznawanie słów.

- Łagodny ubytek słuchu (80-89%): Niewielkie trudności z rozpoznawaniem słów.

- Utrata słuchu w stopniu umiarkowanym (70-79%): Umiarkowane trudności z rozpoznawaniem słów.

- Poważny ubytek słuchu (50-69%): Znaczne trudności z rozpoznawaniem słów.

- Głęboki ubytek słuchu (<50%): Słabe rozpoznawanie słów.

Konfiguracja ubytku słuchu: Wzorzec wyników rozpoznawania słów może dostarczyć informacji na temat konfiguracji ubytku słuchu. Na przykład, jeśli istnieje znacząca różnica w wynikach dla różnych częstotliwości, może to sugerować określony wzór ubytku słuchu.

Wykorzystanie zrównoważonych fonetycznie list słów: Testy audiometrii werbalnej często wykorzystują zrównoważone fonetycznie listy słów, które zawierają reprezentatywną próbkę dźwięków mowy. Pomaga to zapewnić, że test dokładnie odzwierciedla zdolność danej osoby do rozumienia codziennej mowy.

Uwzględnienie kontekstu: Ważne jest, aby wziąć pod uwagę ogólny kontekst zdolności słyszenia danej osoby. Na przykład osoba z lekkim ubytkiem słuchu może nadal dobrze rozpoznawać słowa w cichym otoczeniu, ale może mieć większe trudności w głośnych lub trudnych sytuacjach akustycznych.

Interpretacja wyników badania audiometrii słownej powinna być przeprowadzona przez wykwalifikowanego audiologa lub protetyka słuchu. Weźmie on pod uwagę cały profil audiometryczny, w tym wyniki audiometrii tonalnej i innych odpowiednich testów, aby zapewnić kompleksowe zrozumienie zdolności słuchowych danej osoby. Wyniki pomagają w podejmowaniu decyzji dotyczących potrzeby stosowania aparatów słuchowych, urządzeń wspomagających lub innych interwencji w celu rozwiązania trudności komunikacyjnych związanych z ubytkiem słuchu.

19. Jakie są wyniki otoskopii w przypadku otosklerozy?

Sama otoskopia może nie zapewnić ostatecznych wyników diagnozowania otosklerozy, ponieważ jest to przede wszystkim technika badania klinicznego mająca na celu wizualizację przewodu słuchowego zewnętrznego i błony bębenkowej. Otoskleroza jest zaburzeniem ucha wewnętrznego, które obejmuje nieprawidłowy wzrost kości w uchu środkowym, szczególnie wokół kości strzemiączka, co może prowadzić do utraty słuchu.

Aby zdiagnozować otosklerozę, zwykle stosuje się połączenie oceny klinicznej i testów diagnostycznych. Oto kilka aspektów otoskopii i powiązanych testów, które mogą być istotne w kontekście otosklerozy:

Otoskopia: Podczas otoskopii lekarz bada przewód słuchowy i błonę bębenkową za pomocą otoskopu. Objawy, które można zaobserwować w otoskopii, obejmują normalną lub lekko cofniętą błonę bębenkową, a czasami może być widoczny niebieskawy odcień (objaw Schwartza) z powodu zmian naczyniowych związanych z otosklerozą. Jednak sama otoskopia nie może ostatecznie zdiagnozować otosklerozy.

Audiometria: Audiometria tonalna jest kluczowym badaniem diagnostycznym w przypadku otosklerozy. Osoby z otosklerozą często wykazują charakterystyczny wzór przewodzeniowego ubytku słuchu, szczególnie przy niskich częstotliwościach. Szczelina powietrzno-kostna na audiogramie może wskazywać na otosklerozę, gdy progi przewodnictwa kostnego mieszczą się w normalnym zakresie, ale progi przewodnictwa powietrznego są podwyższone.

Tympanometria: Tympanometria mierzy ruchomość błony bębenkowej i układu ucha środkowego. W przypadku otosklerozy tympanometria może wykazywać charakterystyczny wzorzec, taki jak normalna podatność, ale zmniejszona objętość przewodu słuchowego. Tympanometria, wraz z audiometrią, przyczynia się do rozpoznania otosklerozy.

Badanie odruchu stapedialnego: Badanie odruchu stapedialnego obejmuje pomiar odruchowego skurczu mięśnia stapedius w odpowiedzi na głośne dźwięki. Osoby z otosklerozą mogą mieć nieobecne lub podwyższone progi odruchu stapedialnego.

Badania obrazowe: Techniki obrazowania o wysokiej rozdzielczości, takie jak tomografia komputerowa kości skroniowej, mogą zapewnić szczegółowe obrazy struktur ucha środkowego. Obrazowanie może ujawnić specyficzne cechy związane z otosklerozą, takie jak charakterystyczny wygląd "podwójnego pierścienia" wokół strzemiączka na skanie TK.

Ostatecznie diagnoza otosklerozy jest często stawiana na podstawie połączenia wywiadu klinicznego, badania fizykalnego, testów audiometrycznych i badań obrazowych. Jeśli podejrzewa się otosklerozę, można skonsultować się z otolaryngologiem lub laryngologiem w celu dalszej oceny i leczenia. Interwencja chirurgiczna, taka jak stapedektomia, jest powszechną opcją leczenia osób z objawową otosklerozą.

20. Jakie wszczepialne protezy słuchu można stosować u dzieci?

U dzieci z ubytkiem słuchu można stosować różne wszczepialne protezy słuchu. Wybór konkretnego urządzenia zależy od takich czynników, jak rodzaj i stopień ubytku słuchu, wiek dziecka i czynniki indywidualne. Oto niektóre z wszczepialnych protez słuchu powszechnie stosowanych u dzieci:

1Implanty ślimakowe: Implanty ślimakowe to urządzenia elektroniczne, które są wszczepiane chirurgicznie w celu zapewnienia bezpośredniej stymulacji elektrycznej nerwu słuchowego. Są one odpowiednie dla dzieci ze znacznym lub głębokim niedosłuchem odbiorczym, które nie odnoszą znaczących korzyści z tradycyjnych aparatów słuchowych. Implanty ślimakowe mogą być stosowane u niemowląt i starszych dzieci.

Aparaty słuchowe zakotwiczone w kości (BAHA): Aparaty słuchowe zakotwiczone w kości (BAHA) to chirurgicznie wszczepiane urządzenia, które wykorzystują przewodnictwo kostne do przekazywania wibracji dźwiękowych bezpośrednio do ucha wewnętrznego. Są one odpowiednie dla dzieci z przewodzeniowym lub mieszanym ubytkiem słuchu lub jednostronną głuchotą. Urządzenia BAHA mogą być wszczepiane starszym dzieciom, gdy czaszka dojrzeje, a także istnieją opcje niechirurgicznych rozwiązań BAHA.

Implanty słuchowe pnia mózgu (ABI): Implanty słuchowe pnia mózgu (ABI) to wszczepiane urządzenia przeznaczone dla osób z brakującym lub uszkodzonym nerwem ślimakowym, co może wystąpić w przypadku obustronnego niedoboru nerwu ślimakowego. ABI są częściej stosowane u dzieci z nerwiakowłókniakowatością typu II (NF2).

Implanty ucha środkowego: Implanty ucha środkowego są chirurgicznie umieszczane w uchu środkowym i mają na celu poprawę transmisji dźwięku przez naturalną ścieżkę słuchową. Urządzenia te można rozważyć w przypadku niektórych rodzajów niedosłuchu odbiorczego lub mieszanego u dzieci.

Vibrant Soundbridge (VSB): Vibrant Soundbridge to rodzaj implantu ucha środkowego, który bezpośrednio napędza struktury ucha środkowego. Jest on odpowiedni dla dzieci z niedosłuchem odbiorczym lub mieszanym i może być wszczepiany dzieciom w wieku od 5 lat.

Rozwiązanie Earlens Contact Hearing Solution: Earlens Contact Hearing Solution to niechirurgiczny, sterowany światłem system aparatów słuchowych, który wykorzystuje soczewkę umieszczoną na błonie bębenkowej w celu bezpośredniego wprawiania jej w drgania. System ten jest odpowiedni dla dzieci z lekkim lub średnio ciężkim niedosłuchem odbiorczym.

Wybór wszczepialnej protezy słuchu opiera się na dokładnej ocenie przeprowadzonej przez audiologa, otolaryngologa (laryngologa) i innych odpowiednich pracowników służby zdrowia. Wiek dziecka, profil słuchu, historia medyczna i indywidualne potrzeby są dokładnie brane pod uwagę w celu określenia najbardziej odpowiedniego urządzenia.

Ważne jest, aby rodzice i opiekunowie ściśle współpracowali z zespołem opieki zdrowotnej w celu podejmowania świadomych decyzji dotyczących rodzaju wszczepialnej protezy słuchu, która najlepiej zaspokoi potrzeby dziecka i zapewni optymalne wyniki słuchowe.
